# The allogenic non-proteinogenic amino acid BMAA-based vaccine breaks up the immune tolerance against colorectal cancer

**DOI:** 10.7150/thno.104722

**Published:** 2025-02-10

**Authors:** Baorui Tian, Shijian Wang, Jixuan Ding, Weiao Qu, Chen Zhang, Fangqun Luan, Nan Wang, Yigong Hou, Mengying Suo, Huimin Liu, Yanan Chen, Yanhua Liu, Jie Yan, Jianbo Zhang, Jia Li, Longlong Wang, Yi Shi, Rong Xiang

**Affiliations:** 1 The School of Medicine, Nankai University, 94 Weijin Road, Tianjin 300071, China.; 2 The department of Gastrointestinal Surgery, Second affiliated Hospital of Chongqing Medical University, 288 Tianwen Road, Chongqing 400072, China.

**Keywords:** Tumor vaccine, β-N-methylamino-L-alanine, Colorectal cancer, Nanoparticles, PD-1/PD-L1

## Abstract

The fundamental issue in immunotherapy is the lack of tumor-specific antigens in most types of tumors, leading to immune tolerance. For approximately 85% of patients with microsatellite stable (MSS) colorectal cancer (CRC), the absence of tumor neoantigens results in poor immunotherapy efficacy. Our previous study demonstrated that the misincorporation of non-proteinogenic proline (Pro) analog azetidine-2-carboxylic acid (AZE) could generate mutated proteins that significantly enhance tumor cell antigenicity and anti-tumor immune responses.

**Methods:** To activate more specific anti-tumor immune responses with fewer side effects, we utilized the non-proteinogenic serine (Ser) analog β-N-methylamino-L-alanine (BMAA), which can be misincorporated into proteins as a Ser substitute by seryl tRNA synthetase at an appropriate rate. BMAA misincorporated neoantigens were detected using mass spectrometry (MS), and cancer cell-enriched peptides with high antigenicity were selected in a murine CRC model for the preparation of BMAA-based self-assembling nanoparticles (SAN). Single-cell sequencing was performed to analyze immune responses induced by SAN vaccination combined with a toll-like receptor 7 agonist (TLRa) adjuvant and BMAA treatment.

**Results:** SAN-TLRa vaccination with BMAA treatment induced an anti-tumor immune microenvironment. This combination stimulated the generation of specific CD8^+^ T cells and IgG targeting BMAA misincorporated neoepitopes, ultimately promoting immune activation, tumor suppression, and prolonged survival in the CRC murine model. Additionally, BMAA combined with SAN vaccine significantly enhanced the efficacy of the immune checkpoint inhibitor anti-PD-1 antibody.

**Conclusion:** Our findings provide a promising strategy for artificially introducing neoantigens using BMAA, which can break immune tolerance without disrupting systemic immune balance. This approach offers novel avenues for CRC immunotherapy.

## Introduction

Tumor immunotherapy holds significant importance for cancer treatment 1, 2, such as tumor vaccines 3 and checkpoint inhibitor therapy 4. Advancements in the area, however, have been impeded by several bottlenecks, among which the absence of neoantigens is of vital importance 5, 6. Tumors originate from the accumulation of mutations in normal cells and are accompanied by immune evasion during their formation, often lacking highly immunogenic neoantigens necessary to initiate the anti-tumor immune response 7. A typical example of this is MSS CRC 8. Based on the mutation patterns of CRC, it can be divided into two types: microsatellite instability (MSI) CRC, characterized by a high mutation burden, and MSS CRC, characterized by a low mutation burden 9. Most MSI CRCs originate from mutations in mismatch repair (MMR) genes, providing them with plenty of neoantigens and rich immune cell infiltration, often augmenting immunotherapy efficacy 10. However, MSI CRCs account for only about 15% of all cases, with the majority being MSS CRCs, which often lack strong immunogenic antigens to elicit effective anti-tumor immune responses 11, 12. Therefore, inducing neoantigens in patients with MSS CRC has significant clinical application potential and research significance 13.

Current research suggests that cancer chemotherapy can increase neoantigens by inducing tumor gene mutations, thereby enhancing tumor suppression through combined immunotherapy methods such as immune checkpoint inhibitors 14. However, chemotherapy may lead to irreversible gene mutation accumulation in normal cells, and its associated bone marrow suppression may even reduce the efficacy of tumor immunotherapy 15. Thus, we attempted to induce neoantigens at the protein level through novel immunotherapy approaches. Protein mistranslation has the capability of producing neoantigens 16. Despite the precision in translation due to the extensive evolutionary conservation of aminoacyl-tRNA synthetases (aaRSs) proofreading pathways, some amino acid analogs can be misincorporated into proteins at a low rate 17. Previously, we have confirmed that the misincorporation of the Pro analog AZE in breast cancer can inhibit tumors and activate immunity 18. Given that the misincorporation of amino acid analogs lacks cell type specificity, and due to the high similarity of AZE's side chain with Pro, its misincorporation rate was high, subsequently affecting the function of normal cells, especially in proliferating cells. Therefore, in this study, we chose the Ser analog BMAA for research. The side chain of BMAA is methylamino, with low similarity to the hydroxyl group of the Ser side chain, resulting in a lower misincorporation efficiency 19, 20, thereby reducing the possibility of side effects. Apart from potential neurological effects with long-term intake, BMAA exhibits lower cytotoxicity towards cell lines compared to other amino acid analogs 21. Extensive research has confirmed that BMAA is primarily misincorporated into proteins through seryl-tRNA synthetase in mammals 22, 23. Moreover, in culture media lacking L-Ser, BMAA can be misincorporated stably into human cellular proteins 24. In this study, we further confirmed BMAA's misincorporation in MSS CRC cells, CT26, both* in vivo* and* in vitro*.

Our research indicated that BMAA misincorporation sites were not random. Therefore, personalized therapeutic cancer vaccines based on screened tumor-enriched neoantigens containing BMAA misincorporation, have highly potential treatment methods as they can induce and enhance antigen-specific immune responses 25. The neoantigen vaccines need support with vectors 26 and adjuvants 27, 28. Charge-modified SAN is a new type of vaccine platform with generalizability for neoantigens 29, 30. Compared with formulations of other particles such as poly (lactic-co-glycolic acid), liposomal, or emulsion of oil in water based on squalene (AddaVax™), SAN elicits a notably higher CD8^+^ T cell response and can remodel the tumor microenvironment 31, 32.

In this study, we proposed that BMAA misincorporation can promote anti-tumor immunity and tumor suppression. We identified a tumor-specific neoepitope containing BMAA misincorporation and constructed an SAN vaccine. In the murine model of CRC, we investigated the safety and anti-tumor efficacy of combined BMAA and SAN-TLRa vaccine treatment. Additionally, we explored the epitope specificity of the immune response induced by the combination of BMAA and SAN-TLRa and its therapeutic effects in combination with anti-PD-1 antibodies. This novel CRC immunotherapy method can creatively solve the dilemma of MSS CRC lacking highly immunogenic neoantigens, break immune tolerance, and hold significant therapeutic potential and research value for MSS CRC and theoretically applicable for a broad range of tumor types.

## Results

### BMAA misincorporation causes specific mutated proteome in CRC cells

**Figure 1:**
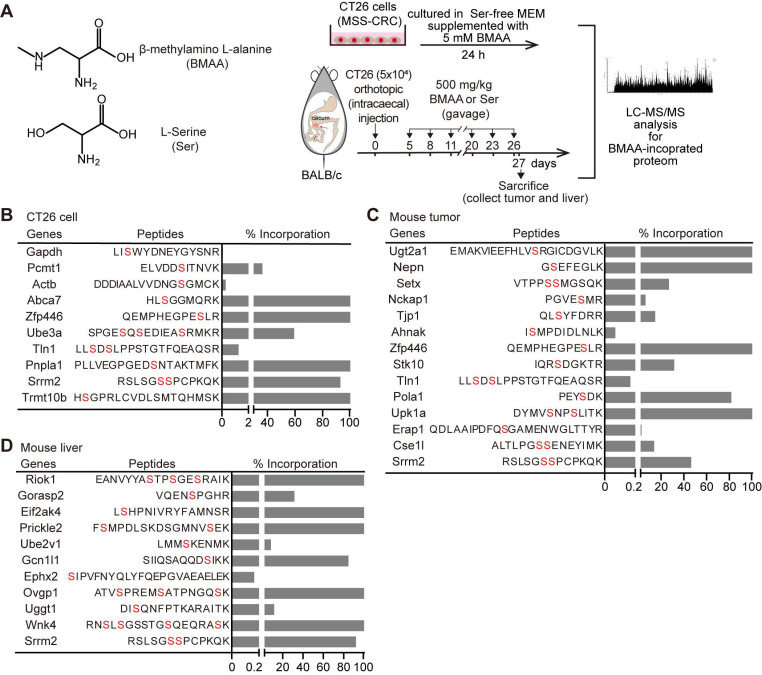
BMAA can be misincorporated into proteins *in vivo* and *in vitro*. (**A**) Structural formulas of the BMAA and Ser, and the scheme depicting the detection of BMAA misincorporation in the Ser position by MS *in vivo* and *in vitro*. (**B-D**) The gene names of BMAA misincorporated proteins, positions of misincorporation, and the proportion of misincorporation in corresponding proteins detected by MS in CT26 cells, mouse tumor, and mouse liver. Alternative locations of BMAA misincorporation were highlighted in red.

BMAA has been mainly reported to substitute Ser in protein synthesis particularly in nerve cells, causing neurological pathologies. To investigate whether BMAA was misincorporated into cancer cells with active protein synthesis, *in vitro*-cultured murine CRC cells CT26 (MSS-CRC) or CT26 allografts in syngeneic BALB/c mice were treated with BMAA followed by LC-MS/MS of the BMAA-misincorporated proteome (Figure 1A). There were 10 (out of 2,308) and 14 (out of 2,722) proteins detected with Ser residues substituted by BMAA in *in vitro* cultured CT26 cells and CT26 allografts respectively (Figure 1B-C). Additionally, we performed repeated analyses to detect BMAA misincorporation in tumor tissues (Figure S1F). In normal liver tissue with active protein synthesis, we observed 11 (out of 2,035) proteins with BMAA misincorporation (Figure 1D). While one common protein, Srrm2, was identified in both normal liver cells and CRC cells, the overall patterns of BMAA-misincorporated proteins differed, likely due to variations in protein expression profiles between the tissues, despite the consistency of misincorporation sites within specific peptide sequences. Furthermore, compared to tumor tissues, the misincorporation of BMAA into proteins was markedly reduced in the kidney, lung, muscle, colon, and lymph nodes, as shown in Figure S1A-E. These results indicated that BMAA could be misincorporated into proteins in CRC cells both* in vitro* and *in vivo* and led to CRC-distinctive BMAA-misincorporated proteome.

### BMAA misincorporation inhibits the growth of CRC in immunocompetent mice

Misincorporation of some non-protein amino acids (NPAs), such as AZE, has been reported to impact cell growth 33. However, the cytotoxicity of NPAs misincorporation depends on the type of NPAs and the proteinogenic amino acid being replaced. The proliferation of *in vitro*-cultured CT26 cells was not affected by BMAA even at the dosage of 2 mM (Figure S2A), which did not affect protein synthesis (Figure S2B). In contrast, the growth of CT26 allografts implanted in syngeneic BALB/c mice was significantly inhibited by intraperitoneal (i.p.) administration of 300 mg/kg body weight of BMAA (Figure S2C-F), suggesting that the anti-tumor effect of BMAA was probably dependent on BMAA misincorporation-caused generation of neoantigen and hence the immune responses against cancer. We further examined the anti-tumor effect of BMAA on CT26 tumors implanted in T lymphocyte-deficient BALB/c nude (nu/nu) mice (Figure S2G), in which BMAA no longer affected the growth of CT26 tumors (Figure S2H-I). These results suggested that BMAA was able to inhibit the CRC growth mainly through T lymphocyte-mediated anti-tumor immune responses.

### The design of SAN CRC vaccine based on BMAA-misincorporated neoepitopes

**Figure 2:**
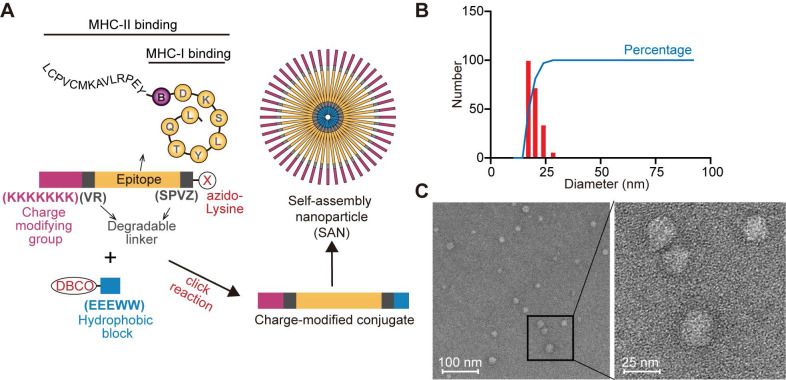
Synthesis, structural, and functional characterization of SAN vaccine based on BMAA misincorporated neoepitope. (**A**) Schematic diagram of modular components comprising charge-modified group, epitope, degradable linker, and hydrophobic block. SAN micelles were formed by click reaction to form the SAN vaccine in ddH_2_O. (**B**) DLS measurement of particle size distribution of SAN vaccine. (**C**) Transmission electron microscopy micrographs of SAN vaccine.

To further enhance specific anti-CRC immune responses against BMAA-misincorporated CRC-specific epitopes, we chose 13 CRC-specific BMAA-misincorporated proteins detected in CRC tumor tissue (Figure 1C) for the design of a peptide vaccine. Among these proteins, CSE1L, ERAP1, POLA1, STK10 showed higher expression in human CRCs compared with normal colon tissues in GEPIA database 34 (Figure S3A). We further analyzed the BMAA-misincorporated peptides (with 10 amino acids flanking both ends included) in these four proteins for their binding affinity with MHC-I in BALB/c mice (H-2-D^d^, H-2-K^d^, and H-2-L^d^) by the Immune Epitope Database (IEDB) algorithm and selected the peptide RPEYSDKSLYTQL of POLA1 with the strongest potential affinity (Table S1). Moreover, we collected tumor tissues and adjacent normal colorectal tissues from CT26 tumor-bearing mice *in vivo* for *Pola1* qPCR experiments. The results showed that the expression of *Pola1* in CRC tissues was markedly elevated compared to the adjacent normal colorectal tissues (Figure S3B). According to the MHC-II peptide binding affinity prediction, we chose the extended peptide LCPVCMKAVLRPEYBDKSLYTQL (B stands for BMAA) as the epitope for the design of a BMAA-misincorporation specific anti-CRC SAN vaccine, in which seven consecutive positively charged lysine residues (KKKKKKK) followed by a degradable linker (VR) at the N-terminal and an azido-modified lysine at the C-terminal by another degradable linker (SPVZ, where Z stands for citrulline) (Figure 2A, Figure S4A-B). The peptide was covalently linked to a hydrophobic module, Dibenzocyclooctyne (DBCO)-EEEWW, which was commercially synthesized and identified (Figure S4C-D), by click-chemistry (Figure 2A). Dynamic light scattering (DLS) measurements indicated that the SANs had a hydrodynamic diameter (HD) between 20 and 30 nm (Figure 2B), which was consistent with the morphology examined by the electron microscope (Figure 2C).

### SAN-TLRa vaccination with BMAA treatment dramatically suppresses tumor growth in the orthotopic murine model of CRC

**Figure 3:**
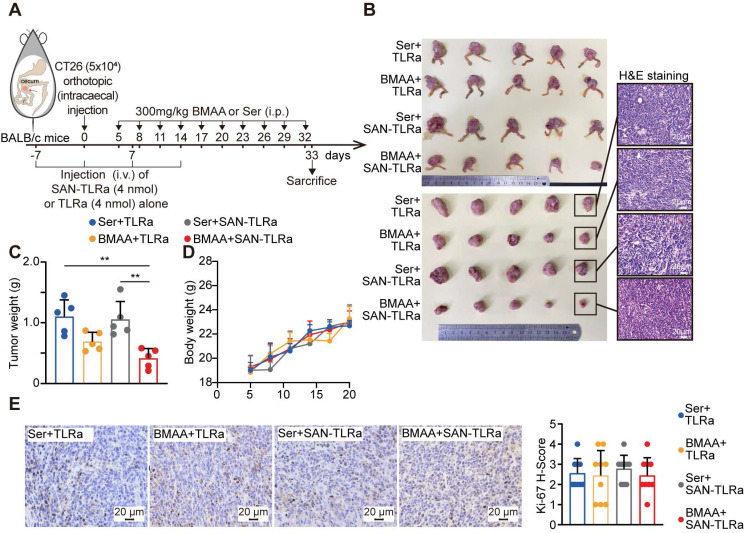
BMAA treatment combined with SAN vaccine demonstrated anti-tumor ability. (**A**) Vaccination and BMAA treatment schedule of CRC treatment assay. BALB/c mice (n = 5) were injected with CT26 cells in the cecum, vaccinated i.v. with TLRa or SAN-TLRa, and administered i.p. with Ser or BMAA. Spleens, tdLNs, tumors, and serum was collected for subsequent experiments at day 33. (**B**) Images of the ceca and tumors shown by H&E staining in mice with indicated treatments. (**C**) Tumor weights of mice with indicated treatments. (**C**) Weight curves of tumor-bearing mice with indicated treatments. (**E**) IHC images and statistical data of Ki-67 in orthotopic tumors of mice with indicated treatments. ^**^P ≤ 0.01, *P* values were calculated by a one-way ANOVA test.

Given that the specific immune microenvironment of the colon may affect the immune responses against tumors triggered by the SAN vaccine, we further investigated its effect in an orthotopic murine model of CRC by injection of CT26 cells at the cecum of BALB/c mice (Figure 3A). Consistently, BMAA treatment alone slightly reduced the tumor burden in the orthotopic CT26 allografts, while SAN vaccination combined with BMAA treatment resulted in a bigger reduction of tumor burden (Figure 3B-D). SAN-TLRa vaccination alone showed no significant effect on tumor growth. The Ki-67 immunohistochemistry (IHC) staining showed that the SAN vaccination and BMAA therapy had no direct impact on the proliferation of cancer cells (Figure 3E), further suggesting the anti-tumor effect of BMAA depended on BMAA-misincorporation induced immune responses.

### SAN-TLRa vaccination with BMAA treatment induces an anti-tumor immune microenvironment

**Figure 4:**
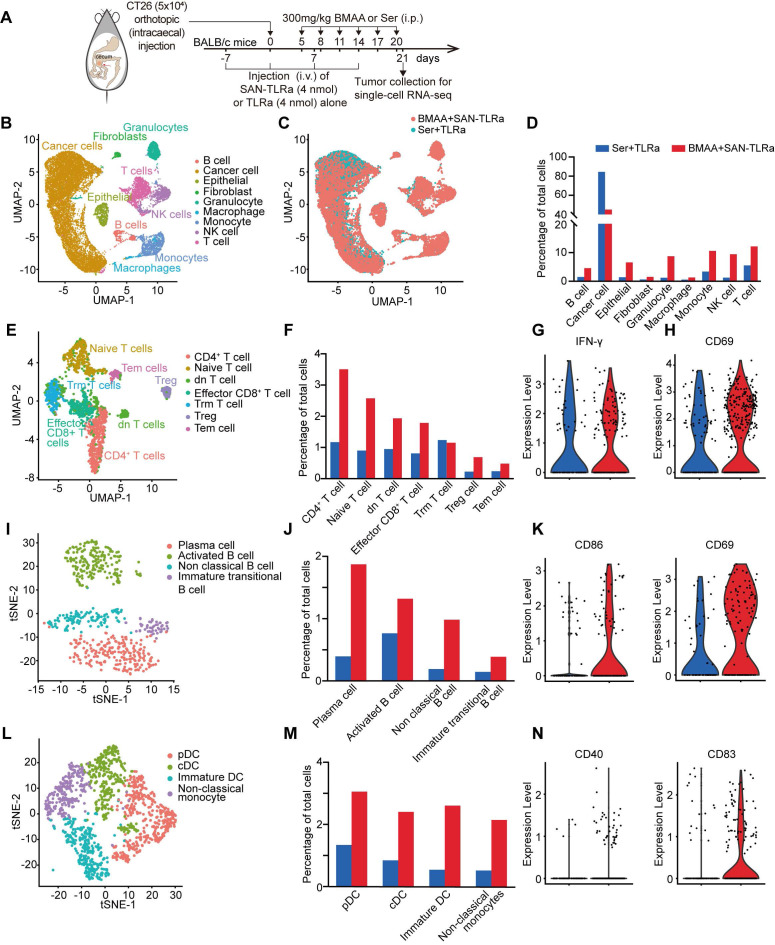
BMAA treatment combined with SAN vaccine promoted an anti-tumor immune microenvironment. (**A**) Vaccination and BMAA treatment schedule of single cell sequencing. BALB/c mice were injected with CT26 cells in the cecum, vaccinated i.v. with TLRa or SAN-TLRa, and administered i.p. with Ser or BMAA. Tumors were collected for single cell sequencing at day 21. (**B**) UMAP representation of single cell sequencing landscape. (**C**) UMAP representation of all cellular clusters showing differences between mice receiving BMAA+SAN-TLRa and Ser+TLRa. (**D**) The fraction of all cellular clusters for the BMAA+SAN-TLRa group (red) and Ser+TLRa group (blue). (**E**) UMAP representation of all T cell clusters from scRNA-seq data. (**F**) The fraction of each T cell subtype among total cells. (**G**) Violin plot of activation-related gene *IFN-γ* in effector CD8^+^ T cells. (**H**) Violin plot of activation-related gene *CD69* in effector CD4^+^ T cells. (**I**) tSNE representation of all B cell clusters from scRNA-seq data. (**J**) The fraction of each B cell subtype among total cells. (**K**) Violin plots of activation-related genes *CD86* and *CD69* in activated B cells. (**L**) tSNE representation of all monocyte clusters from scRNA-seq data. (**M**) The fraction of each monocyte subtype among total cells. (**N**) Violin plots of activation-related genes *CD40* and *CD83* in cDCs.

To get insights into the mechanisms of vaccine efficacy, we performed single cell transcriptome analysis in the orthotopic CT26 CRC murine model receiving SAN-TLRa and BMAA (experimental group) or TLRa with Ser (control group) treatment (Figure 4A, Figure S5A-G). The results showed significant variations in the makeup of immune cells infiltrating tumors between the experimental group and control group (Figure 4B-4C). Relative to the control group, the recruitment of various immune cells in the tumors of mice who received SAN-TLRa and BMAA treatment was highly activated (Figure 4D). Within the T cell subpopulations, except for tissue-resident memory (Trm) T cells, recruitment by all other clusters of T cells into the tumors in the experimental group was increased compared with the control group (Figure 4E-F). In the SAN-TLRa and BMAA treatment group, CD8^+^ T cells exhibited upregulation of the activation-related gene *IFN-γ* (Figure 4G), and a similar activation-related gene, CD69, was detected in CD4^+^ T cells (Figure 4H). Four clusters of B cells were identified, and compared with the control group, cells from these four B cell subpopulations were enriched in the experimental group (Figure 4I-J), with activated B cells showing increased expression of activation-related genes *CD86* and *CD69* (Figure 4K). Monocytes were divided into four clusters, each of which was upregulated in the SAN-TLRa and BMAA treatment group compared with the control group (Figure 4L-M), and conventional dendritic cells (cDCs) also showed upregulation of the activation-related genes *CD40* and *CD83* (Figure 4N).

These data indicated that SAN-TLRa and BMAA induced the efficient recruitment of various immune cells to tumors in a highly activated state.

### SAN-TLRa vaccination with BMAA treatment effectively boosts the infiltration of CD8^+^ cytotoxic T-lymphocyte (CTL) against CRC

We next investigated the impacts of BMAA on the CRC immune microenvironment. In the murine model of CRC by subcutaneously implanted CT26 (Figure S2C), flow cytometry analysis of the spleen immune cells showed that the BMAA treatment resulted in increased populations of CD8^+^ T cells (Figure S6A) and migratory dendritic cells (DCs, CD80^+^ CD86^+^) (Figure S6B), while it showed no significant effects on macrophages, NK cells, or others (Figure S6C-I).

**Figure 5:**
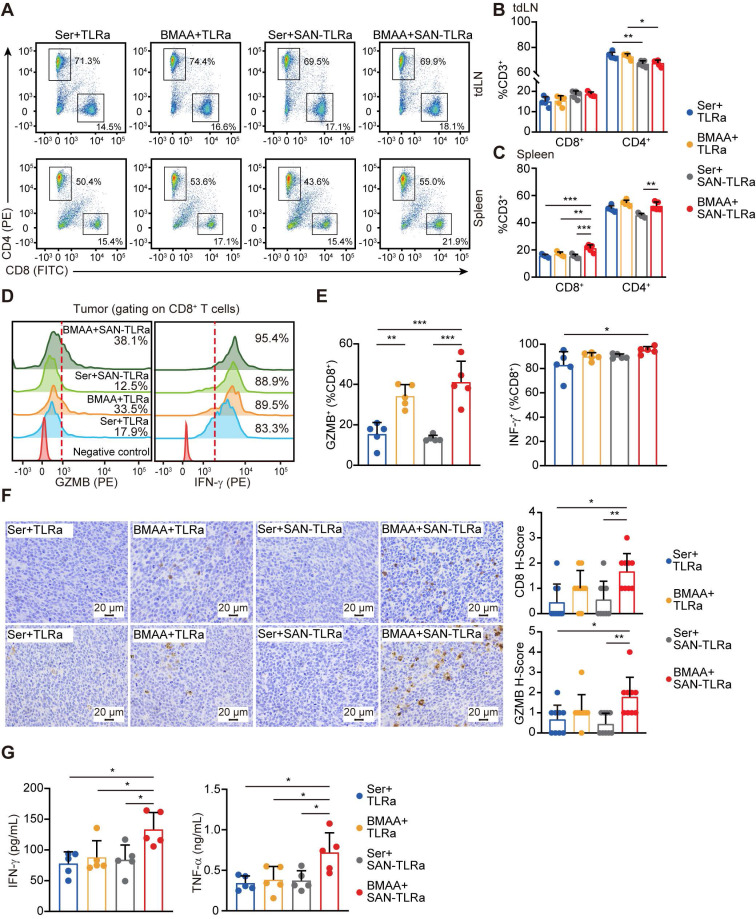
BMAA treatment combined with SAN vaccine activated cytotoxic CD8^+^ T cells. (**A**) Plots of CD8^+^ T cells and CD4^+^ T cells in tdLNs and spleens of mice with indicated treatments. (**B**) Percentage of CD8^+^ and CD4^+^ subpopulations for CD3^+^ cells in tdLNs. (**C**) Percentage of CD8^+^ and CD4^+^ subpopulations for CD3^+^ cells in spleens. (**D**) Histograms of GzmB^+^ or IFN-γ^+^ CD8^+^ T cells in tumors of mice with indicated treatments. (**E**) Percentages of GzmB^+^ or IFN-γ^+^ subpopulations of tumor-infiltrating CD8^+^ cells. (**F**) IHC images and statistical data of CD8 and GzmB within orthotopic tumor tissues of mice with indicated treatments. (**G**) IFN-γ and TNF-α content in tumor lysates of mice with indicated treatments. The mice (n = 5) followed the same schedule as shown in Figure 3a. ^*^P ≤ 0.05, ^**^P ≤ 0.01, ^***^P ≤ 0.001, *P* values were calculated by a one-way ANOVA test in **B**,** C**,** E**,** G**, and Kruskal-Wallis test in **F**.

We further analyzed the proportions of CD8^+^ T cells in draining lymph nodes (dLNs), spleens and tumors of the orthotopic murine model of CRC by flow cytometry. As shown in Figure 5A-C, SAN-TLRa vaccination followed by BMAA treatment efficiently increased the populations of CD8^+^ T cells in both dLNs and spleens. Within the tumor tissues, more CD8^+^ T cells were activated by SAN-TLRa vaccination and BMAA treatment, shown by the increased Granzyme B^+^ (GzmB^+^) and IFN-γ^+^ CD8^+^ T cells (Figure 5D-E). The infiltration of the cytotoxic CD8^+^ T cells was also evaluated with looking at the levels of CD8 and GzmB by IHC (Figure 5F) which revealed that treatment with BMAA enhanced by SAN-TLRa led to higher expression of CD8 and GzmB.

To further confirm the BMAA-induced anti-tumor immune responses, we isolated tumor tissues and analyzed the generation of pro-inflammatory cytokines by ELISA. Consistently, increased IFN-γ and TNF-α production was observed in SAN-TLRa-vaccinated and BMAA-treated mice (Figure 5G).

### DC function was elicited by BMAA combined with SAN-TLRa favorably

**Figure 6:**
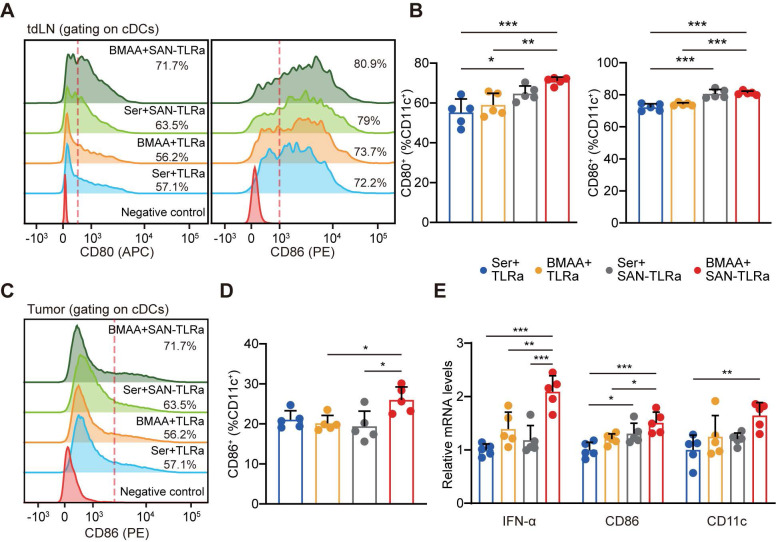
BMAA treatment combined with SAN vaccine enhanced DC function. (**A**) Histograms of CD80^+^ or CD86^+^ cells in tdLNs of mice with indicated treatments. (**B**) Percentage of CD80^+^ or CD86^+^ subpopulation for CD11c^+^ cells in tdLNs. (**C**) Histograms of CD86^+^ cells in tumors of mice with indicated treatments. (**D**) Percentages of CD86^+^ subpopulation of CD11c^+^ cells in tumors. (**E**) *IFN-α*, *CD80*, and *CD11c* expression in tdLNs of mice with indicated treatments. The mice (n = 5) followed the same schedule as shown in Figure 3a. ^*^P ≤ 0.05, ^**^P ≤ 0.01, ^***^P ≤ 0.001, *P* values were calculated by a one-way ANOVA test.

DCs play an active role in anti-tumor effects by presenting tumor antigens and priming anti-tumor T cells 35. To evaluate whether SAN-TLRa vaccination promoted DC maturation, we used markers such as the co-stimulatory molecules CD80 and CD86. In the tumor-draining lymph nodes (tdLNs), SAN-TLRa vaccination slightly increased the proportion of CD80^+^ and CD86^+^ DCs, while SAN-TLRa vaccination followed by BMAA treatment further enhanced the DC maturation (Figure 6A-B). Consistently, we also observed significant increase in the CD86^+^ DCs population after the treatment of SAN-TLRa together with BMAA in the tumor tissue (Figure 6C-D). To further confirm the immunoactivity of DCs, we examined the expression of mature DCs-associated molecules, i.e., *IFN-α*, *CD86* and *CD11c* by RT-qPCR in the dLNs. The results showed that SAN-TLRa followed by BMAA treatment boosted the expression of *IFN-α*, *CD86* and *CD11c* compared with other control groups (Figure 6E).

### SAN-TLRa induces BMAA-misincorporation-specific anti-CRC immune responses

**Figure 7:**
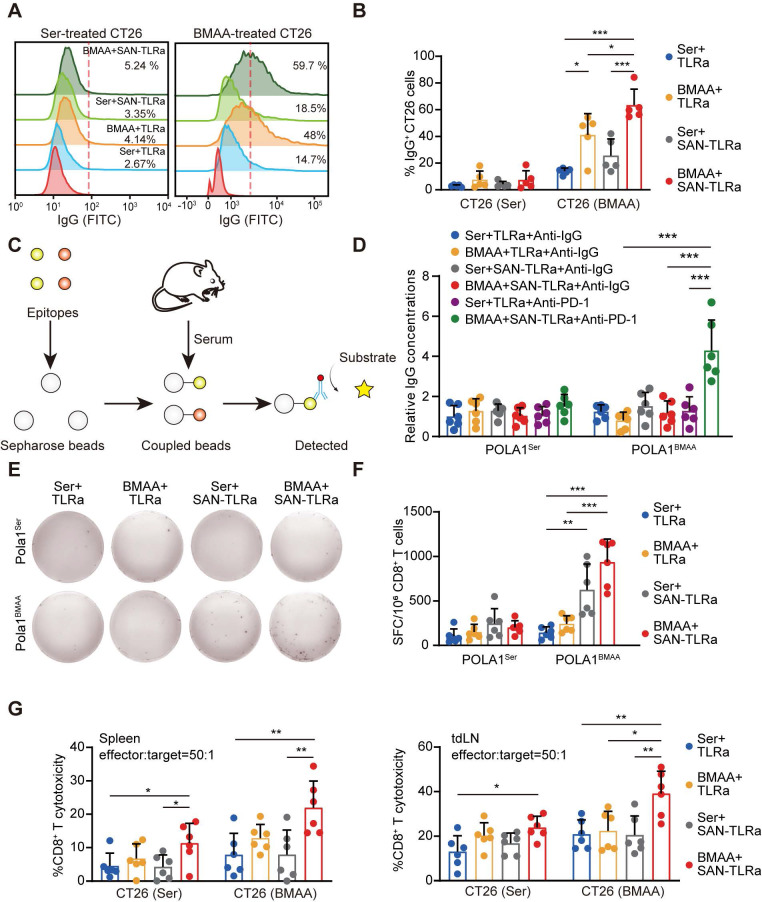
BMAA treatment combined with SAN vaccine generated neoantigen-specific antibodies and CD8^+^ T cells. (**A**) Histograms of IgG^+^ CT26 cells after incubation with sera of mice with indicated treatments, which followed the same schedule as shown in Figure 3a (n = 5). (**B**) Percentages of IgG^+^ subpopulations in CT26 cells treated with either 5 mM BMAA or 5 mM Ser. (**C**) Schematic diagram of epitope-specific IgG detection. (**D**) The level of IgG antibody specific to BMAA misincorporated neoepitope POLA1^BMAA^ and WT epitope POLA1^Ser^ in sera of mice (n = 6) followed the same schedule as shown in Figure 8b. (**E**) IFN-γ ELISpot response of CD8^+^ T cells in mouse spleens specific to BMAA misincorporated neoepitope POLA1^BMAA^ and WT epitope POLA1^Ser^. (**F**) Quantification of spot-forming cells (SFC) per 10^6^ CD8^+^ T cells. (**G**) The cytotoxicity of CD8^+^ T cells in mouse spleens and tdLNs toward CT26 cells treated with 5 mM Ser or 5 mM BMAA. The mice in **E-G** followed the same schedule as shown in Figure 4a (n = 6). ^*^P ≤ 0.05, ^**^P ≤ 0.01, ^***^P ≤ 0.001, *P* values were calculated by a one-way ANOVA test.

To measure BMAA-misincorporation-specific IgG, CT26 cells were treated with BMAA or Ser *in vitro* to detect BMAA-misincorporated neoantigen-specific IgG in the serum of mice vaccinated with SAN-TLRa and BMAA treatment. BMAA markedly increased the specific IgG targeting BMAA-misincorporated CT26 cells, which was enhanced by SAN-TLRa (Figure 7A-B). We further detected the production of neoepitope-specific IgG (Figure 7C). The combination of SAN-TLRa and BMAA treatment did not generate new epitope-specific IgG. However, following the combination of PD-1 antibodies, the levels of epitope-specific IgG in the serum significantly increased (Figure 7D). To gain insights into the antigen specificity of treated mice, we assessed the reactivity of the CD8^+^ T cells in spleens to BMAA-misincorporated neoepitope. We observed IFN-γ release by CD8^+^ T cells derived from the BMAA+ SAN-TLRa group stimulated with BMAA-misincorporated neoepitope instead of WT epitope, demonstrating that BMAA combined with SAN-TLRa stimulates the production of antigen-specific CD8^+^ T cells (Figure 7E-F). The CD8^+^ T cell cytotoxicity was then evaluated by the lactate dehydrogenase (LDH) cytotoxicity assay. CD8^+^ T cell-mediated killing toward tumor cells, especially the BMAA-treated cells in BMAA+ SAN-TLRa group was significantly increased compared with other groups (Figure 7G). Taken together, our results supported the ability of SAN-TLRa to elicit a CTL response and the production of neoepitope-specific IgG specifically to BMAA-misincorporated neoepitope.

### Biosafety and autoimmune functions are investigated with BMAA+ SAN-TLRa

To assess the toxicity of BMAA and SAN-TLRa, BMAA or Ser combined with SAN-TLRa or TLRa were administered to non-tumor bearing mice (Figure S7A). Body weight measurements indicated no significant effects from the application of BMAA or SAN-TLRa (Figure S7B). The blood smears and bone marrow smears, routine blood examinations and blood biochemistry analysis also revealed no abnormalities (Figure S7C-D, Table S2). H&E and CD8 IHC staining indicated that BMAA and SAN-TLRa had no significant adverse effects on vital organs (Figure S7E, 8A-B).

### Survival and efficacy are markedly enhanced after BMAA+ SAN-TLRa combined with anti-PD-1 antibody administration

**Figure 8:**
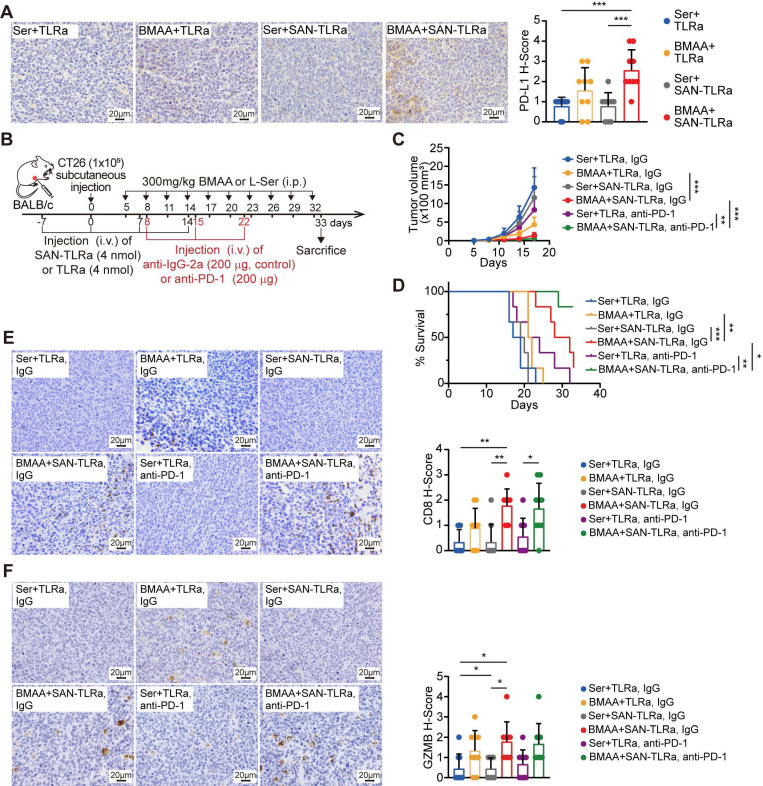
BMAA+SAN-TLRa showed better efficacy and survival outcomes combined with anti-PD-1 antibody administration. (**A**) IHC images and statistical data of PD-L1 in mouse tumors. The mice followed the same schedule as shown in Figure 3a. (**B**) BALB/c mice (n = 6) were injected with CT26 cells subcutaneously, vaccinated i.v. with TLRa or SAN-TLRa, and administered i.p. with Ser or BMAA, and administered i.p. with anti-IgG-2a or anti-PD-1. Tumors were collected for subsequent experiments on day 33. (**C**) Tumor growth curve using tumor volume data from mice with indicated treatments. (**D**) Kaplan-Meier survival curve of mice with indicated treatments. (**E**) IHC staining and quantification of CD8^+^ cells within the tumors of mice with indicated treatments. (**F**) IHC staining and quantification of GzmB^+^ cells within the tumors of mice with indicated treatments. ^*^P ≤ 0.05, ^**^P ≤ 0.01, ^***^P ≤ 0.001, *P* values were calculated by a one-way ANOVA test in **A**,** E**,** F**, two-way ANOVA test in **C**, and log-rank test in **D**.

Mechanistically, BMAA can break the immune tolerance induced by low immunogenicity of CRC, so BMAA has the therapeutic potential alongside immune checkpoint inhibitors. We analyzed the expression of inhibitory receptor PD-L1 in tumor tissues, canonical markers of T cell suppression. The administration of BMAA and SAN-TLRa correlated with higher levels of PD-L1 in the tumors (Figure 8A). The single cell sequencing and qPCR results also demonstrated that the expression of immune checkpoint receptors in tumor cells was upregulated in the BMAA+ SAN-TLRa group compared with the control group (Figure S9A-B). Therefore, we assessed the efficacy of BMAA+ SAN-TLRa combined with anti-PD-1 in subcutaneous CT26 murine model (Figure 8B). Results demonstrated that BMAA+ SAN-TLRa combined with anti-PD-1 could markedly improve mouse survival and inhibit tumor growth, and even eliminate half of the tumors (Figure 8C-D). IHC assay was employed to evaluate the capability of combined anti-PD-1 antibody to enhance cytotoxic CD8^+^ T cell function (Figure 8E-F).

## Discussion

Immunotherapy relies on the immune system and aims to unblock the obstructed pathways in anti-tumor immune responses 36. However, for patients with MSS CRC, there is often a lack of highly immunogenic neoantigens 13. Currently, there are no effective therapies to increase neoantigens with high immunogenicity. Under normal conditions, about 15% of body cells exhibit protein mistranslation 37. Tumor cells proliferate rapidly with vigorous protein synthesis 38. Therefore, the probability of protein mistranslation in tumor cells is higher 39. Thus, we hypothesized that by introducing amino acid analogs that do not naturally exist in normal organisms 40, neoantigens could be generated to enhance tumor immunogenicity and activate anti-tumor immune responses. Different from neoantigens generated by mutations 41, we explored the effect of artificially introduced neoantigens generated by BMAA misincorporation. We were heartened by the activation of DC and CD8^+^ T cell responses against the BMAA-misincorporated neoepitope in an orthotopic CT26 murine model.

Previously, we showed that misincorporation of the Pro analog AZE can promote anti-tumor immune responses by increasing the immunogenicity of mistranslated proteins 18. Due to the high structural similarity between AZE and Pro 42, 43, AZE has shown a high rate of misincorporation and toxicity. We therefore chose BMAA, whose methylamino side chain is more distinct from the side chain of Ser, for further research in this study. In fact, the misincorporation rate of BMAA is significantly lower than the rate of AZE, leading to lower associated toxicity. We found specificity of misincorporation of BMAA for Ser through MS both *in vitro* and *in vivo*. BMAA misincorporation occurred only at specific Ser sites for unique peptides, as detected in various experiments. Moreover, the strong metabolism of tumors results in the ingestion of more BMAA than vital organs. After detecting the codons of misincorporation sites, we found that misincorporation was not specific to any particular codon of Ser. Ongoing efforts are needed to focused on unraveling the mechanism of the specificity of misincorporation sites to create neopeptides. We can't limit the misincorporation of BMAA to the tumor area in the tumor area, but we can boost specific immune responses by the development of cancer vaccines targeting BMAA misincorporated neoantigens in tumor.

MSS CRC poses significant challenges for immunotherapy due to its lack of immunogenic neoantigens. Current approaches, such as personalized vaccines 44 and gut microbiome modulation 45, are often limited by high costs and narrow applicability. This study introduces a cost-effective and broadly applicable strategy leveraging BMAA-induced neoantigens to enhance tumor immunogenicity. Combined with a self-assembling nanoparticle vaccine and TLR7 agonist, this method elicits robust CD8^+^ T cell responses and synergizes with immune checkpoint inhibitors, demonstrating strong tumor suppression and extended survival in preclinical models. This approach offers a scalable solution for MSS CRC and other low-immunogenic tumors. BMAA does not exist in normal human tissues 46, 47, so peptides misincorporated with BMAA specifically in tumor sites possess higher specificity. The treatment of BMAA provides new insights for finding anti-tumor vaccine targets. Furthermore, the relatively simple and cost-effective process makes it an attractive option for creating 48, 49. In addition to anti-tumor immunity targeting the BMAA misincorporated proteins, a potential advantage of the vaccine is epitope spreading 50, 51. As a novel peptide-based vaccine platform, SAN has been used to explore the potential of using amino acid analogs to create a personalized tumor vaccine. To enhance the tumor specificity of the vaccine, we initially screened proteins that were overexpressed in CRC compared to normal colon tissue as candidates. There is a lack of MHC binding affinity prediction for amino acid analogs, so we screened the epitopes of vaccines based on original peptides without BMAA misincorporation. Considering the limited accuracy of MS detection, we did not select epitopes based on the percentage of incorporation. In this study, we chose the commonly used and well-validated TLR agonist Resiquimod as an adjuvant. For tumors with low immunogenicity, immunotherapy combined with BMAA and SAN-TLRa can elicit anti-tumor immunity more effectively. Considering the rapid growth of CT26 cells in the murine model used in our study, it is challenging to achieve vaccine efficacy post-tumor establishment before humane euthanasia is necessary. Therefore, we initiated vaccine injections one week prior to tumor cell inoculation, and subsequent optimization of the tumor model will validate the efficacy of vaccine injections starting one week after tumor establishment. In this study, we primarily detected immune function on day 33, when differences in tumors were significantly observed. In fact, single cell sequencing performed on day 21 revealed a greater degree of immune activation with the combination of BMAA and SAN-TLRa. Future studies could monitor immune function at additional time points, such as 7 and 14 days. We could expand the quality and quantity of the specific T cell responses for BMAA misincorporated neoepitopes by combining it with immune checkpoint inhibitor anti-PD-1 antibodies. Additionally, we observed the generation of antibodies specific to the vaccine epitope following the combined treatment of BMAA and SAN-TLRa with anti-PD-1 antibodies, and further experimental testing is required to characterize these antibodies. Immunotherapy combined with BMAA and SAN-TLRa has the potential to play a critical role in the therapeutic efficacy of combination immunotherapies in many tumors. Finally, the treatment combined with BMAA and SAN-TLRa may be extended to other amino acid analogs and neoepitopes, and a further method for screening amino acid analogs and misincorporated epitopes needs to be developed for more effective treatments.

Altogether, we demonstrated that the amino acid analog BMAA can improve the immunogenicity of CRC by misincorporating into proteins. BMAA elicited tumor suppression and anti-tumor immune responses, primarily involving CD8^+^ T cells and DCs, which could be further enhanced by SAN-TLRa and anti-PD-1 antibodies. Treatment with BMAA combined with SAN-TLRa induced specific IgG and CD8^+^ T cells against the BMAA misincorporated neoepitope. Furthermore, the administration of either BMAA or SAN-TLRa did not produce significant toxic side effects in mice. We proposed a novel immunotherapy approach for CRC that can creatively address the challenge of MSS CRC lacking highly immunogenic neoantigens, thereby breaking immune tolerance. This approach holds significant therapeutic potential and research value for the immunotherapy of MSS CRC and other tumors without highly immunogenic neoantigens.

## Materials and methods

### Cell lines

The cell line CT26 was purchased from the American Type Culture Collection (ATCC). All cell lines were cultured in Dulbecco's modified Eagle's medium (DMEM, Biological Industries, Kibbutz Beit-Haemek, Israel) supplemented with 100 μg/mL streptomycin, 100 U/mL penicillin (Biological Industries), and 10% fetal bovine serum (FBS, Biological Industries) in a humidified atmosphere at 37 °C with 5% CO_2_. For BMAA-related experiments* in vitro*, Ser-free minimal essential medium (MEM, Biological Industries, Israel) was used.

### Mice

BALB/c female mice (6 weeks-old) and BALB/c female nude mice (6 weeks old) were purchased from the Jackson Laboratory. All animal experiments were approved by the Animal Care and Use Committee at Nankai University (Tianjin, China). BALB/c mice were kept on custom research diets containing low (0 IU/kg) levels of Ser (Jiangsu Xietong, Nanjing, China).

Orthotopic and subcutaneous CRC models were used in this study. Briefly, a subcutaneous CRC model was established by injection of 1.0 × 10^5^ tumor cells into the right flanks of the mice. Tumor volume was calculated with the formula: (width^2^ × length)/2. For survival analysis, survival time was defined as time required for the tumor volume to reach 1,500 mm^3^. In the orthotopic CRC model, mice were anesthetized with 1% sodium pentobarbital. After shaving and disinfection of the abdomen, mice underwent laparotomy. 5.0 × 10^4^ CT26 cells in 25 µL Matrigel (Sigma-Aldrich, St Louis, MO, USA) were injected into the cecum wall.

In this study, BMAA (Shaoyuan Chemical Technology, Shanghai, China) or Ser (Solarbio Science & Technology Co., Ltd., Beijing, China) was administered i.p. or by oral gavage. For vaccination, mice were injected intravenously (i.v.) with SAN vaccines pre-mixed with TLRa (resiquimod, MCE, USA) or TLRa alone as a control in 50 μL ddH_2_O. Mice were vaccinated three times with an interval of one week between vaccinations, the first of which started seven days before tumor challenge. Mouse anti-PD-1 antibody (BioXcell, West Lebanon, NH, USA) or mouse anti-IgG2a antibody (BioXcell) was administered i.p. at 200 µg/mouse three times (one injection every seven days).

### Detection of BMAA misincorporation

Tissues or cells were harvested and snap frozen on dry ice, and MS was performed by Beijing Baitai Parker Biotechnology Co., Ltd. for the detection of BMAA misincorporation.

### Preparation of SAN

MHC-I and MHC-II peptide binding affinity predictions were performed at the IEDB Tools website 52. After determining the neoepitope, we designed the charge-modified neoepitope and hydrophobic block based on previous research experience 29, which were synthesized by WuXi AppTec (Shanghai, China). The purity (≥95%) and identity of the final compound were verified through high-performance liquid chromatography (HPLC) and MS. The conjugate SAN vaccine was produced by linking the charge-modified neoepitope to the hydrophobic block using a copper-free strain-promoted azide-alkyne cycloaddition click chemistry reaction in DMSO at 40°C for 16 h. DLS was used to characterize the diameter of the SAN vaccine using a Laser light scattering system (BI-200SM laser light scattering instrument, Brookhaven, USA). Nanoparticle morphology was investigated by transmission electron microscopy (Talos L120C G2, Thermo Fisher Scientific, USA).

### Cell proliferation assay

CT26 cells were seeded in 96-well plates at a density of 300 cells per well. The cell numbers from three wells in each group were counted every day using Countess II (Thermo Fisher Scientific).

### Single cell RNA-seq analysis

For the single-cell analysis, tumor samples from three mice with identical treatment conditions were pooled, and equal volumes of their tumor tissues were used to prepare the sequencing samples. The tumor tissues were collected for single cell suspension preparation using the 10x Genomics Chromium Controller. Following the assessment of cell viability (viability >85%), the process continued with reverse transcription, complementary DNA (cDNA) amplification, and library creation according to the guidelines provided by the manufacturer. Quality-approved libraries were sequenced on an Illumina platform, and data analysis was conducted using Cell Ranger. The workflow for scRNA-seq data processing is detailed in Figure S6.

### Tissue processing

To obtain a single cell suspension of lymphocytes or splenocytes, spleens or lymph nodes harvested from mice were ground with a syringe plunger and filtered through a 40 μm nylon mesh with cold PBS containing 2% FBS. Tumor tissues were digested with collagenase type IV (1 mg/mL, Solebao), DNase I (200 μg/mL, Solebao), and hyaluronidase (100 μg/mL, Solebao) for 30 min at 37 °C, followed by passing through 40 μm nylon mesh to obtain single cell suspension. Contaminating red blood cells were then lysed using red blood cell lysis buffer (Solebao). For the IFN-γ enzyme-linked immune absorbent spot (ELISpot) assay and LDH cytotoxicity assay, CD8^+^ T cells were purified by MojoSort ™ Mouse CD8 T Cell Isolation Kit (BioLegend).

### Flow cytometry

Antibodies utilized for flow cytometry are detailed in Table S3. Cells were stained in the dark on ice for 1 h with an antibody cocktail, then washed twice with 1 mL cold PBS, and resuspended in 500 μL PBS. For intracellular staining, cells were fixed with 1% paraformaldehyde (Sigma) and permeabilized with Perm/Wash™ buffer (BD Biosciences) after extracellular staining. The permeabilized cells were stained with intracellular cytokine antibodies on ice for 1 h in Perm/Wash™ buffer.

### Staining of tissue sections

Tumor and other organ tissues were fixed in 4% paraformaldehyde for 48 h, and paraffin-embedded subsequently. Specimens embedded in paraffin blocks were cut into 6 μm sections. Sections were deparaffinized and rehydrated. Thereafter, the tissue sections were stained with hematoxylin and eosin (H&E, Solebao). For IHC staining, after antigen retrieval by boiling with sodium citrate buffer (Solebao), sections were incubated with primary antibody (Table S3) overnight at 4 °C. The sections were stained with biotinylated secondary antibodies and streptavidin-HRP, followed by incubation with diaminobenzidine (DAB) solution and hematoxylin counterstain. Nine images were captured randomly in each group for statistical analysis. IHC scores were computed by combining the intensity score and the percentage of area positively stained as mentioned before 18.

### Enzyme-linked immunosorbent assay (ELISA)

Serum was collected from whole blood centrifuged at 2,000 × g. Tumor tissues were lysed in RIPA buffer (Solebao) supplemented with 1 × protease inhibitor cocktail (Solebao) for 30 min on ice. Lysates were centrifuged at 12,000 × g to collect the supernatants. According to the manufacturer's protocols, the concentrations of TNF-α, IFN-β, IL-2, and IL-6 in serum and tumor supernatants were measured by ELISA (Dldevelop, Wuxi, China).

### Quantitative real-time reverse transcription-PCR (qRT-PCR)

Total RNA was extracted from tissues or cells using TRIzol reagent (Yeasen Biotech, Shanghai, China), and cDNA was synthesized with the reverse transcription kit (Yeasen). Real-time quantitative PCR was performed using SYBR Green SuperMix (Yeasen) on the LightCycler96 system (Roche, Mannheim, Germany). The sequences of all primers used in this study are listed in Table S3. Transcript levels of each target gene were normalized to β-actin expression level.

### Tumor-specific IgG analysis

CT26 cells were treated with either 5 mM BMAA or 5 mM Ser for 24 h, and then incubated in 5% (v/v) serum for 1 h on ice. After washing, cells were incubated with Alexa Fluor 488-conjugated anti-mouse IgG (1:100, BioLegend) for 1 h on ice. Washed cells were analyzed by flow cytometry.

### LDH cytotoxicity assay

Purified CD8^+^ T cells from spleens and tdLNs were activated by 1 μg/mL CD3e and 2 μg/mL CD28 monoclonal antibody (eBioscience). 2.5 × 10^5^ activated CD8^+^ T cells were incubated with 5 × 10^3^ CT26 tumor cells treated with either 5 mM BMAA or 5 mM Ser for 2 h. The supernatant was centrifuged and analyzed by LDH cytotoxicity assay using the CyQuant™ LDH Cytotoxicity Assay Kit (Invitrogen). The percentage of cytotoxicity was determined using the following formula: % Cytotoxicity = [(LDH release from the experiment - Spontaneous LDH release by target cells - Spontaneous LDH release by effector cells) / (Total maximum LDH release - Spontaneous LDH release)] × 100%.

### IFN-γ ELISpot assay

1 × 10^5^ purified mouse CD8^+^ T cells were cultured with 10 μg/mL peptides in serum-free culture medium (Dakewe, Shenzhen, China, 6015012) for 24 h. Antigen-specific cells were detected by Mouse IFN-γ precoated ELISpot kit (Dakewe). Spots were then counted by Dakewe Biotech Co., Ltd. The number of peptide-specific spots per well was converted into the number of spots per million cells.

### Morphology, routine blood tests and biochemical tests

Blood was extracted from the vena cava of euthanized mice. Routine blood parameters were detected by Tianjin Genink Biotechnology Co., Ltd. Blood biochemistry was analyzed by biochemical autoanalyzer (MNCHIP, Tianjin, China). The morphology of blood and bone marrow was observed by Giemsa (Solarbio) stained smears using an Olympus BX 51 microscope (Olympus Corporation, Japan).

### Puromycin incorporation analysis

Global translation was indicated by puromycin incorporation analysis using O-propargyl-puromycin (OPP, MCE). For the incorporation of OPP into newly synthesized proteins, cells were labelled with 10 μM OPP for 2 h. Then the azide-biotin compound was conjugated to an alkyne group on OPP by a click reaction. After incubation with HRP-streptavidin, the signals were visualized using a chemiluminescence detection kit (Epizyme Biotech, Shanghai, China).

### Statistical analysis

All quantitative data were shown as mean ± standard error of the mean (SEM), and the normal distribution was analyzed initially for all data sets by the Kolmogorov-Smirnov and Shapiro-Wilk normality tests. If the data sets met the normal distribution, the groups were compared by Student's t-test or analysis of variance (ANOVA); if not, nonparametric tests were used to compare the differences. We used Tukey's Multiple Comparisons Test for multiple comparisons analysis following ANOVA. Survival curves were constructed using the Kaplan-Meier method and analyzed using the log-rank test. Statistical analysis was performed using GraphPad Prism 8.0 software (San Diego, CA). A value of *P* ≤ 0.05 (^*^P ≤ 0.05, ^**^P ≤ 0.01, ^***^P ≤ 0.001) was considered significant.

## Supplementary Material

Supplementary figures and tables.
