# Supplementary material for: The allogenic non-proteinogenic amino acid BMAA-based vaccine breaks up the immune tolerance against colorectal cancer
Source: Theranostics. 2025 Feb 10;15(7):3143–58. doi: 10.7150/thno.104722 (PMC11898291; doi:10.7150/thno.104722)

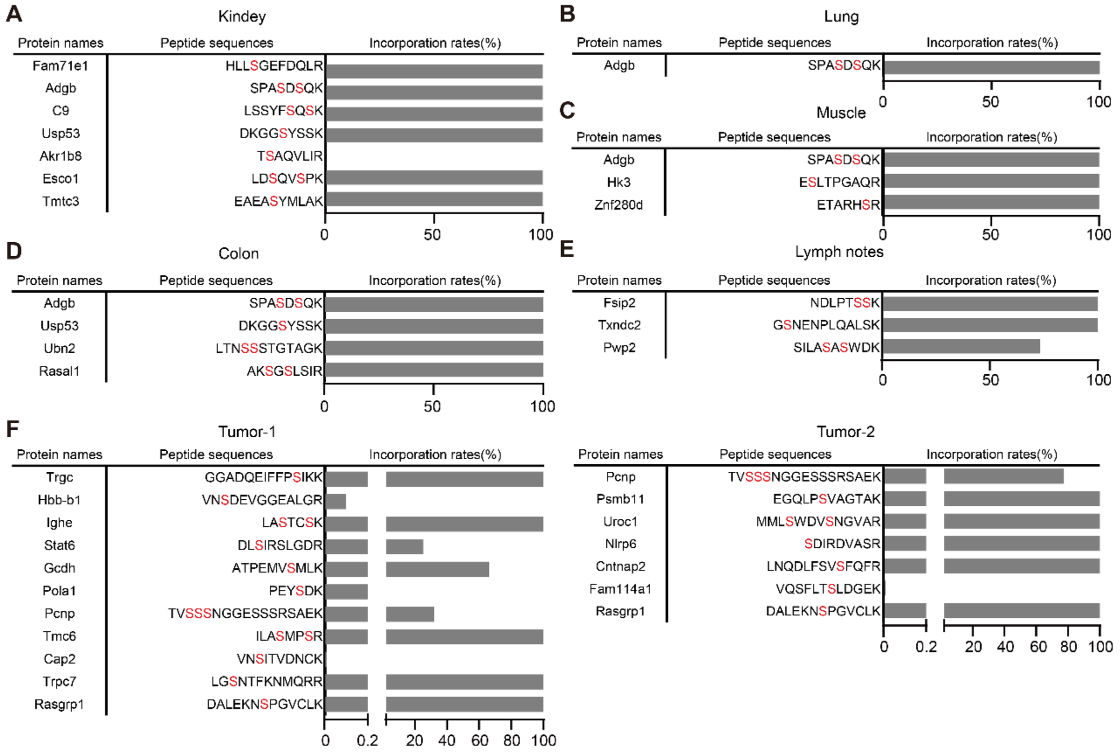

Figure S1 BMAA misincorporation into proteins across different mouse tissues. (**A**) Proteins with BMAA substitution and their relative misincorporation rates in kidney. (**B**) Quantitative analysis of misincorporation sites in lung proteins. (**C**) Detected BMAA-modified proteins and their misincorporation proportions in muscle. (**D**) Distribution and frequency of BMAA substitutions in colonic proteins. (**E**) Identification and quantification of BMAA misincorporation in lymphatic tissue. (**F**) Repeated detection of BMAA misincorporation in tumor tissues.

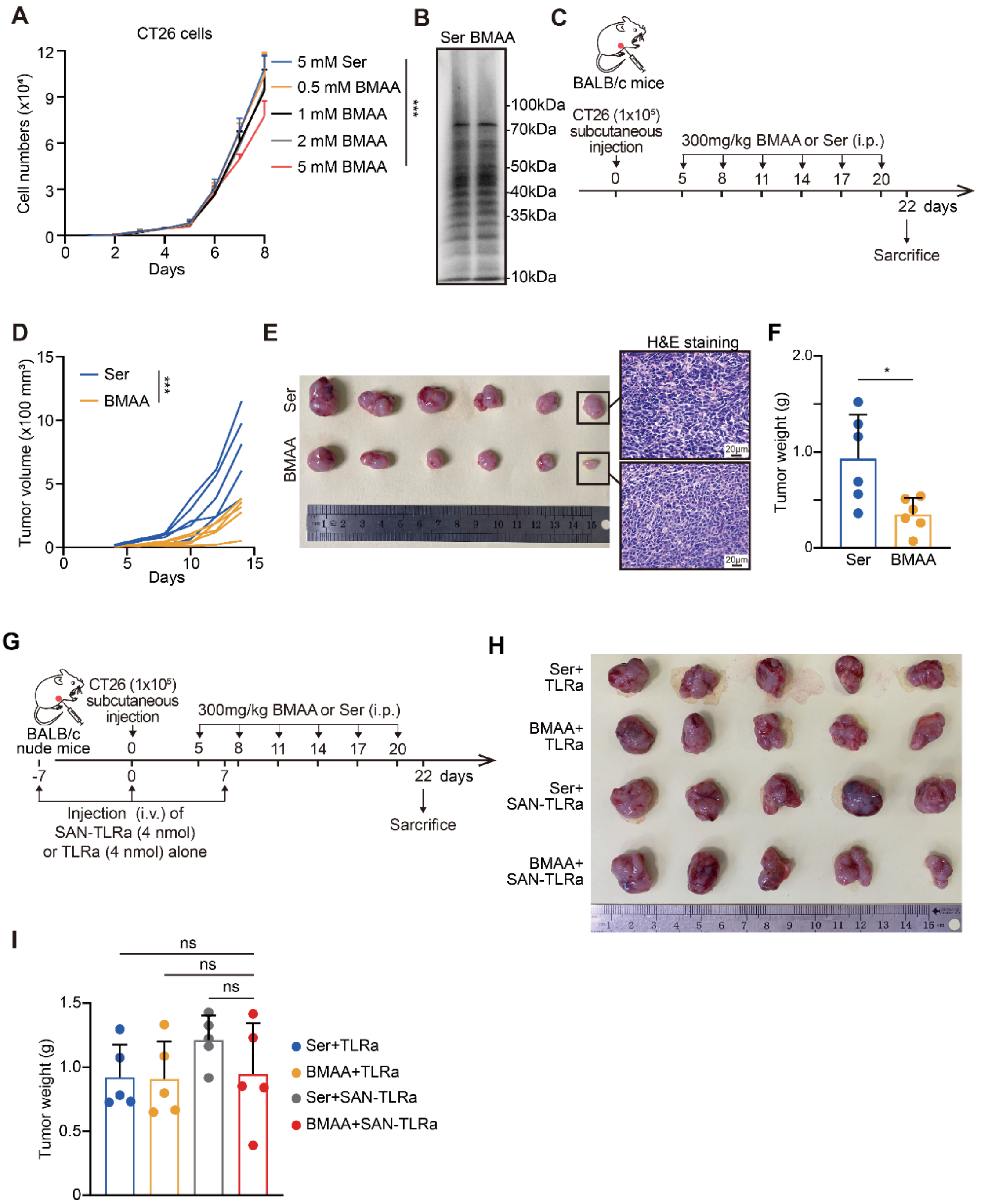

Figure S2 BMAA was able to inhibit CRC growth mainly through T lymphocyte-mediated anti-tumor immune responses. (**A**) The cell number curve of CT26 cells treated with Ser concentration of 5 mM, or BMAA concentration of 0.5 mM, 1 mM, 2 mM, 5 mM (n = 3). (**B**) Global translation in CT26 cells treated with 5 mM Ser or BMAA for 24 h. (**C**) BALB/c mice (n = 6) were injected with CT26

cells subcutaneously, and administered i.p. with Ser or BMAA. Tumors and spleens were collected on day 22 for subsequent experiments. (**D**) Tumor growth curve of mice treated with Ser or BMAA. (**E**) Images of tumors shown by H&E staining in mice with indicated treatments. (**F**) The weights of the tumor allografts of mice treated with Ser or BMAA. (**G**) BALB/c nude mice (n = 5) were injected with CT26 cells subcutaneously, intravenously injected with TLRa or SAN-TLRa, and intraperitoneally injected with Ser or BMAA. Tumors were collected for photography and weighing on day 22. (**H**) Tumor images from mice with indicated treatments. (**I**) The tumor weights of nude mice with indicated treatments. $^{*}P \leq 0.05$, $^{***}P \leq 0.001$, P values were calculated by a one-way ANOVA test in **i**, two-way ANOVA test in **A**, **D**, and two-tailed unpaired Student's t test in **F**.

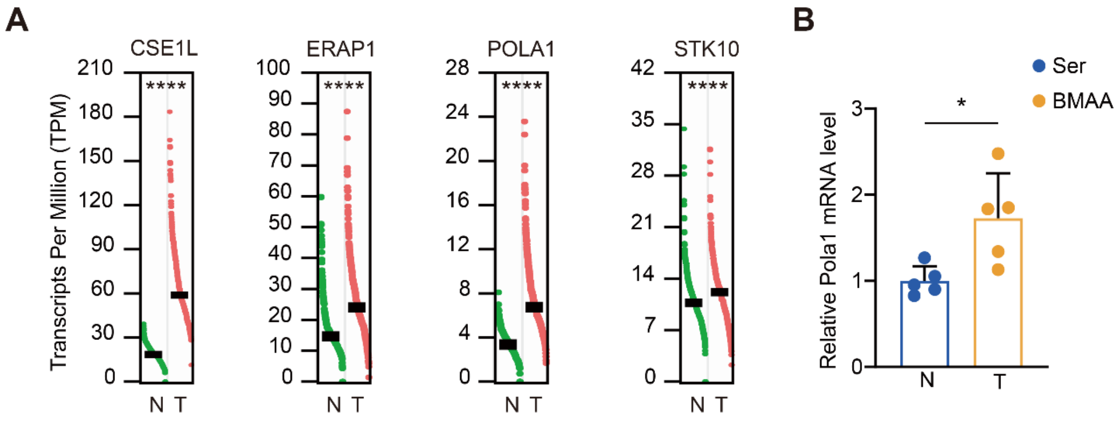

Figure S3 *Pola1* was highly expressed in CRC relative to normal colorectal

tissue. (**A**) Corresponding 4 genes high expression in CRC relative to normal colorectal tissues analyzed with GEPIA, which detected the presence of BMAA misincorporation as shown in Figure 1c. (**B**) *Pola1* expression in tumor and normal colorectal tissue of BALB/c mice with CT26 orthotopic injection. $^{*}P \leq 0.05$, $^{****}P \leq 0.0001$, *P* values were calculated by two-tailed unpaired Student's t test in **B**.

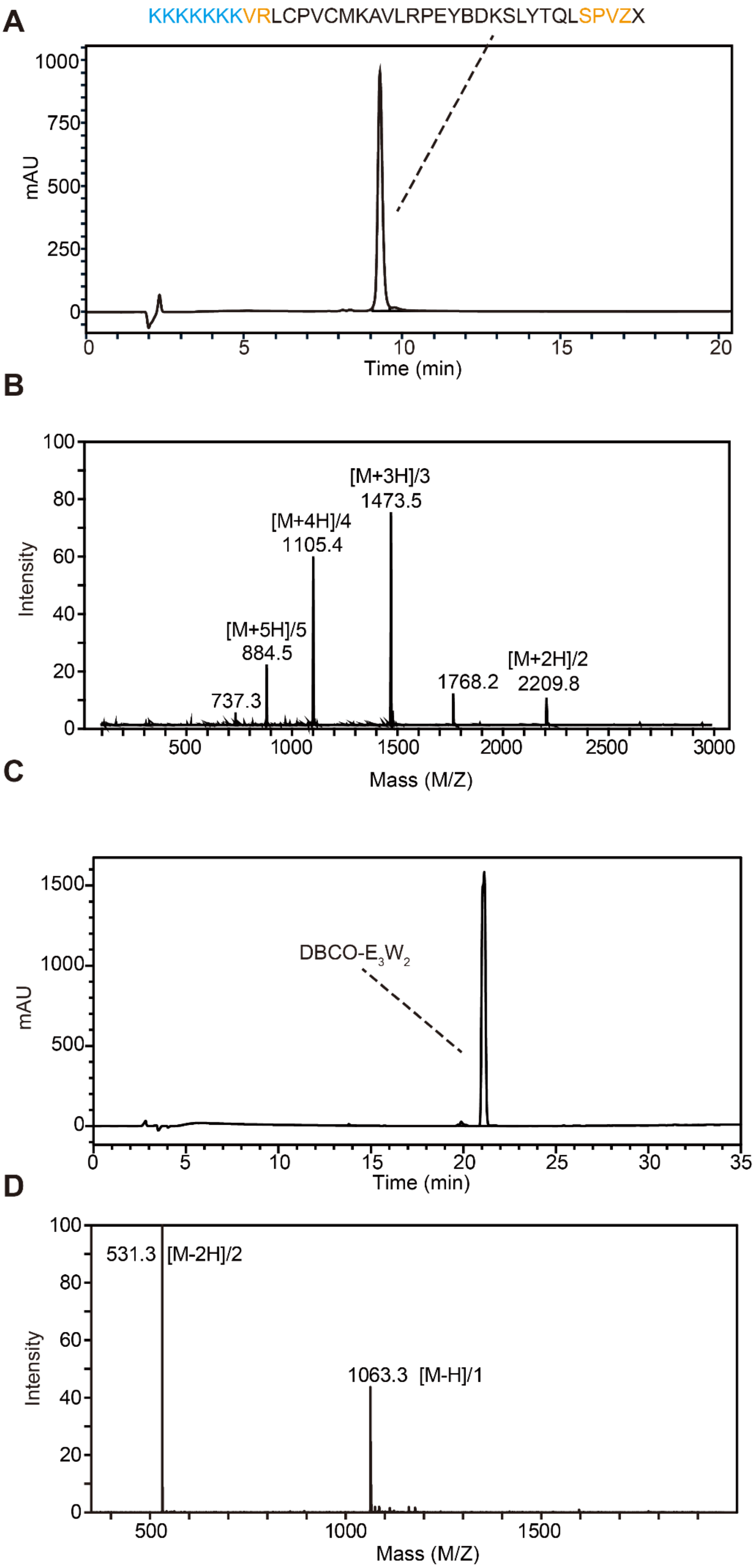

A
KKKKKKKVRLCPVCMKAVLRPEYBDKSLYTQLSPVZX
mAU
Time (min)
B
Intensity
[M+3H]/3
1473.5
[M+4H]/4
1105.4
[M+5H]/5
884.5
737.3
1768.2
[M+2H]/2
2209.8
Mass (M/Z)
C
mAU
DBCO-E3W2
Time (min)
D
Intensity
531.3 [M-2H]/2
1063.3 [M-H]/1
Mass (M/Z)

Figure S4 The identification of SAN modular components by HPLC-MS. (**A**) HPLC analysis to assess the purity of the hydrophobic block. (**B**) MS identification of the hydrophobic block. (**C**) HPLC analysis to assess the purity of the charge-modified neoepitope. (**D**) MS identification of the charge-modified neoepitope.

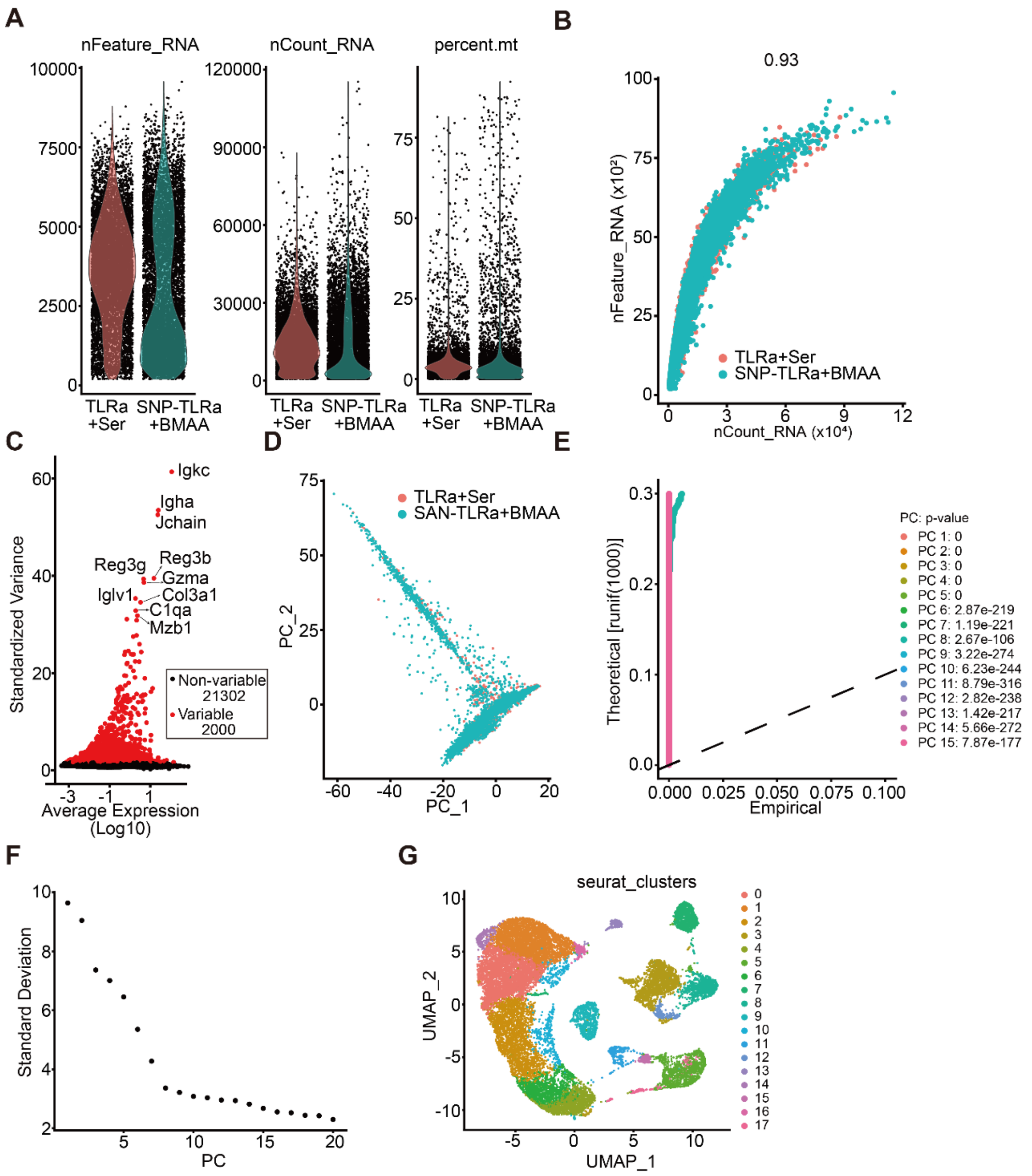

Figure S5 Processing of scRNA-seq data from mouse tumors. (**A**) Assessment of gene and molecule detections per cell revealed variations in gene count (nFeature_RNA), molecular count (nCount_RNA), and mitochondrial gene percentage (percent.mt). (**B**) Detected gene numbers correlated strongly with sequencing depth (Pearson's r = 0.93). (**C**) Variance analysis across 23,302 genes in tumor cells highlighted 10 highly variable genes (red dots) against a

backdrop of non-variable genes (black dots). (**D**) Dimensionality reduction via PCA simplified data analysis. (**E, F**) PCA identified 15 principal components significantly influencing variance (P < 0.05). (**G**) UMAP clustering visualized 18 distinct cell populations in primary tumors, enhancing understanding of tumor heterogeneity.

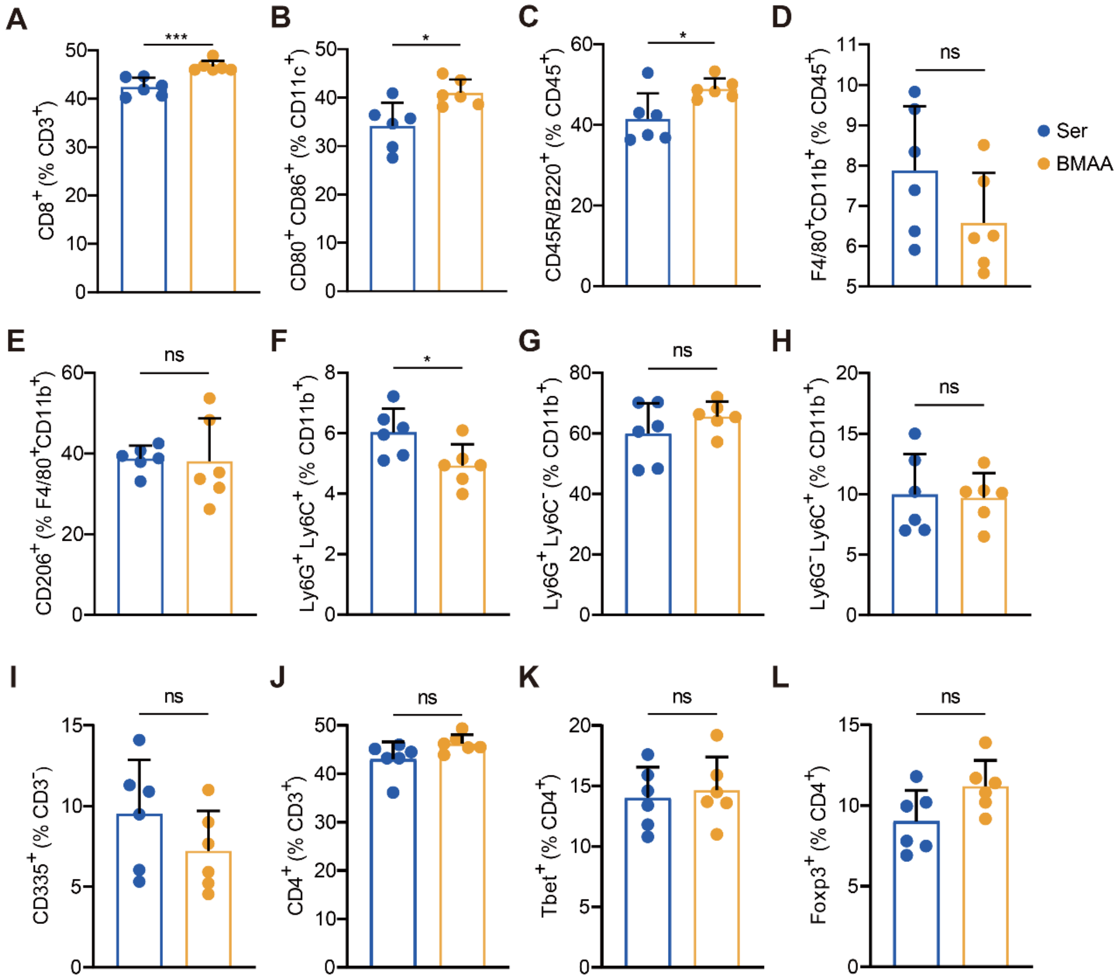

Figure S6 The effect of BMAA treatment on the proportion of immune cells in

the spleen. The mice (n = 6) followed the same schedule as shown in Figure S1C. Spleens were collected and single cells were isolated for flow cytometry. (**A**) The proportion of the $CD8^+$ subpopulation of $CD3^+$ cells to detect $CD8^+$ T cells. (**B**) The proportion of the $CD80^+$ $CD86^+$ subpopulation of $CD11c^+$ cells to detect DCs. (**C**) The proportion of the $CD45R/B220^+$ subpopulation of $CD45^+$ cells to detect B cells. (**D**) The proportion of the $F4/80^+$ $CD11b^+$ subpopulation of $CD45^+$ cells to detect macrophages. (**E**) The proportion of the $CD206^+$ subpopulation of $F4/80^+$ $CD11b^+$ cells to detect M1 and M2 macrophages. (**F**) The proportion of the $Ly6G^+$ $Ly6C^+$ subpopulation of $CD11b^+$ cells to detect neutrophils. (**G**) The proportion of the $Ly6G^+$ $Ly6C^-$ subpopulation of $CD11b^+$ cells to detect neutrophils. (**H**) The proportion of the $Ly6G^-$ $Ly6C^+$ subpopulation of $CD11b^+$ cells to detect monocytes. (**I**) The proportion of the $CD335^+$ subpopulation of $CD3^-$ cells to detect NK cells. (**J**) The proportion of the $CD4^+$ subpopulation of $CD3^-$ cells to detect $CD4^+$ T cells. (**K**) The proportion of the $Tbet^+$ subpopulation of $CD4^+$ cells to detect T-helper 1 cells. (**L**) The proportion of the $Foxp3^+$ subpopulation of $CD4^+$ cells to detect regulatory T cells. $^*P \leq 0.05$, $^{***}P \leq 0.001$, P values were calculated by two-tailed unpaired Student's t test.

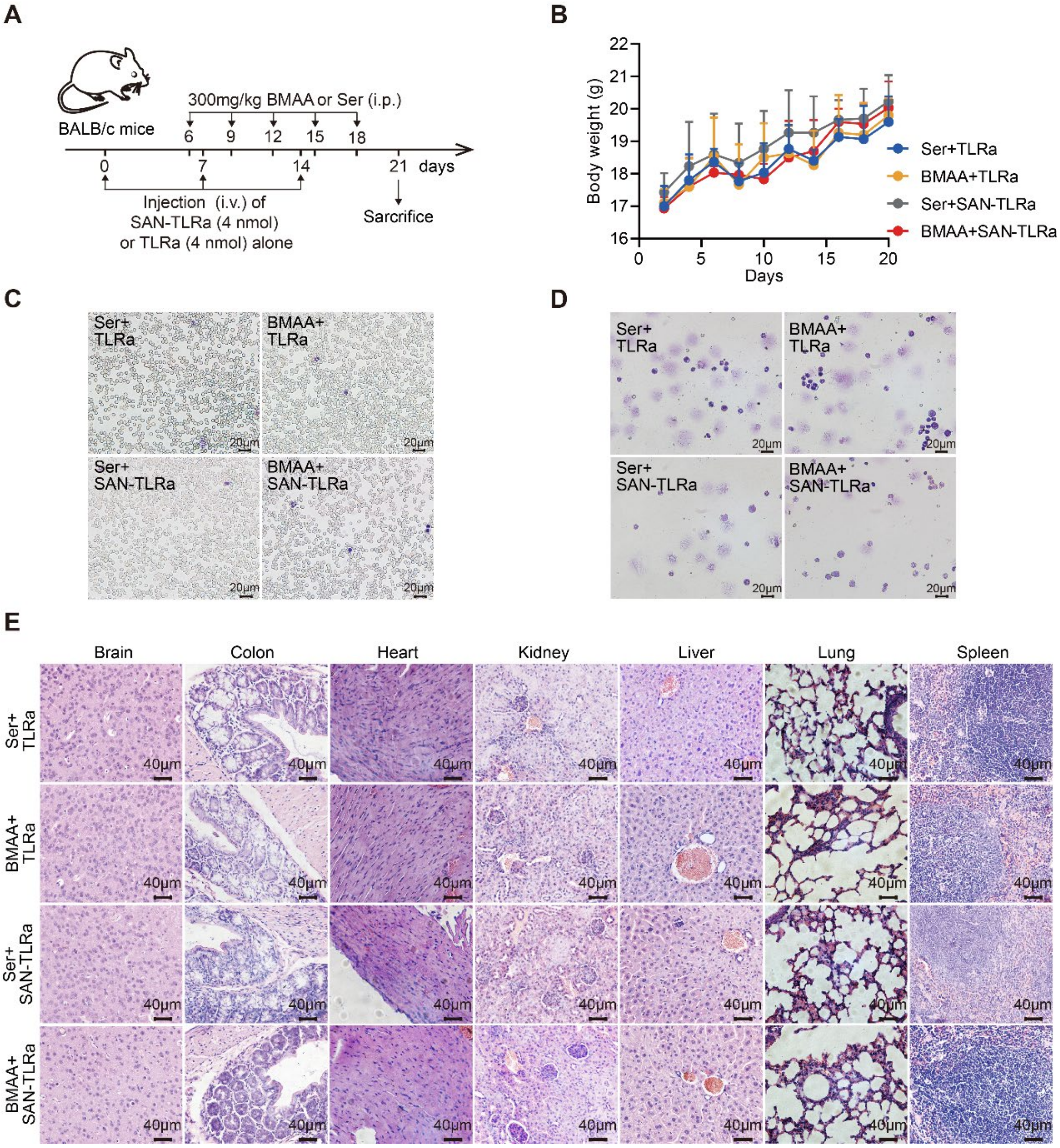

Figure S7 BMAA administration or SAN-TLRa vaccination had no significant toxicity. (**A**) Vaccination and BMAA administration schedule of toxicity assay. BALB/c mice (n = 3) were administered i.v. with TLRa or SAN-TLRa, and i.p. with Ser or BMAA. Blood, bone marrow, and vital organs of mice were collected for toxicity assay on day 21. (**B**) Weight curve of non-tumor-bearing mice with indicated treatments. (**C**) Cell morphology in the blood of non-tumor-bearing mice with indicated treatments. (**D**) Cell morphology in bone marrow of non-

tumor-bearing mice with indicated treatments. (**E**) H&E staining images of vital organs in non-tumor-bearing mice with indicated treatments.

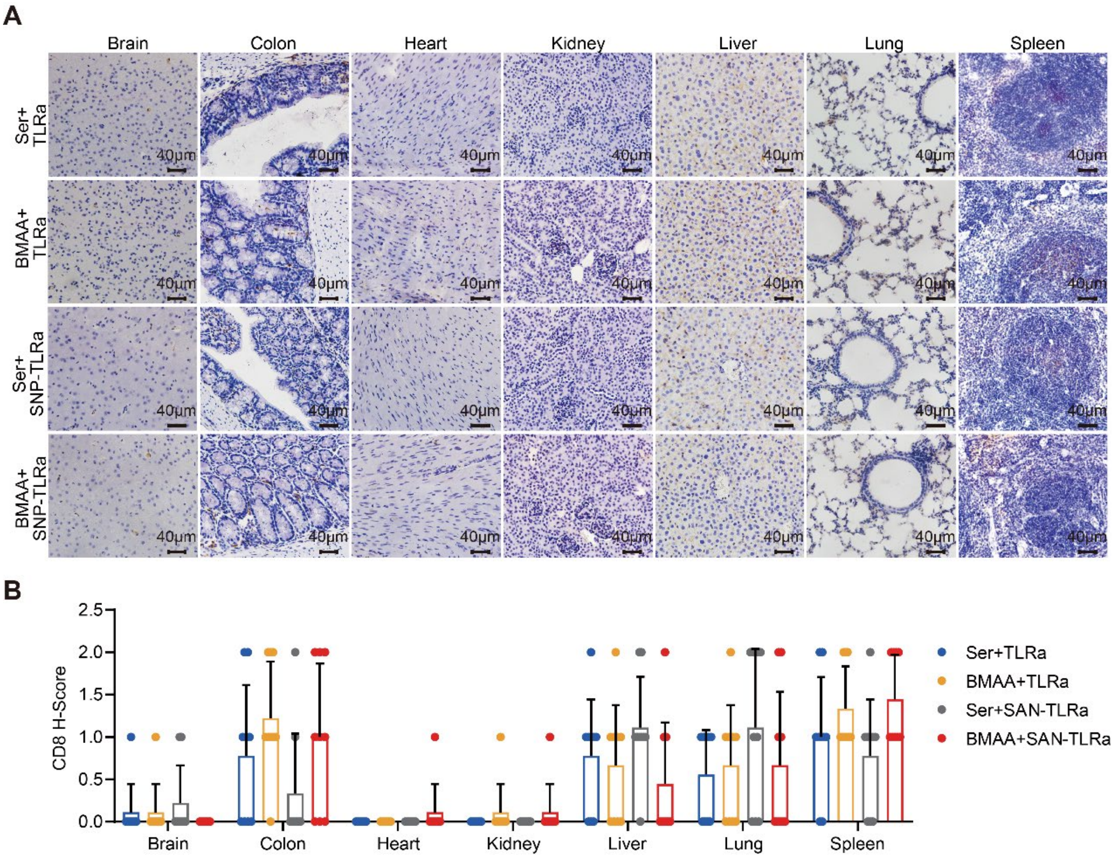

Figure S8 BMAA or SAN-TLRa had no significant effect on CD8^{+} T cells in vital organs. The mice followed the same schedule as shown in Figure S1C. IHC images of CD8 and statistical data in vital organs of non-tumor-bearing mice with indicated treatments. *P* values were calculated by a one-way ANOVA test.

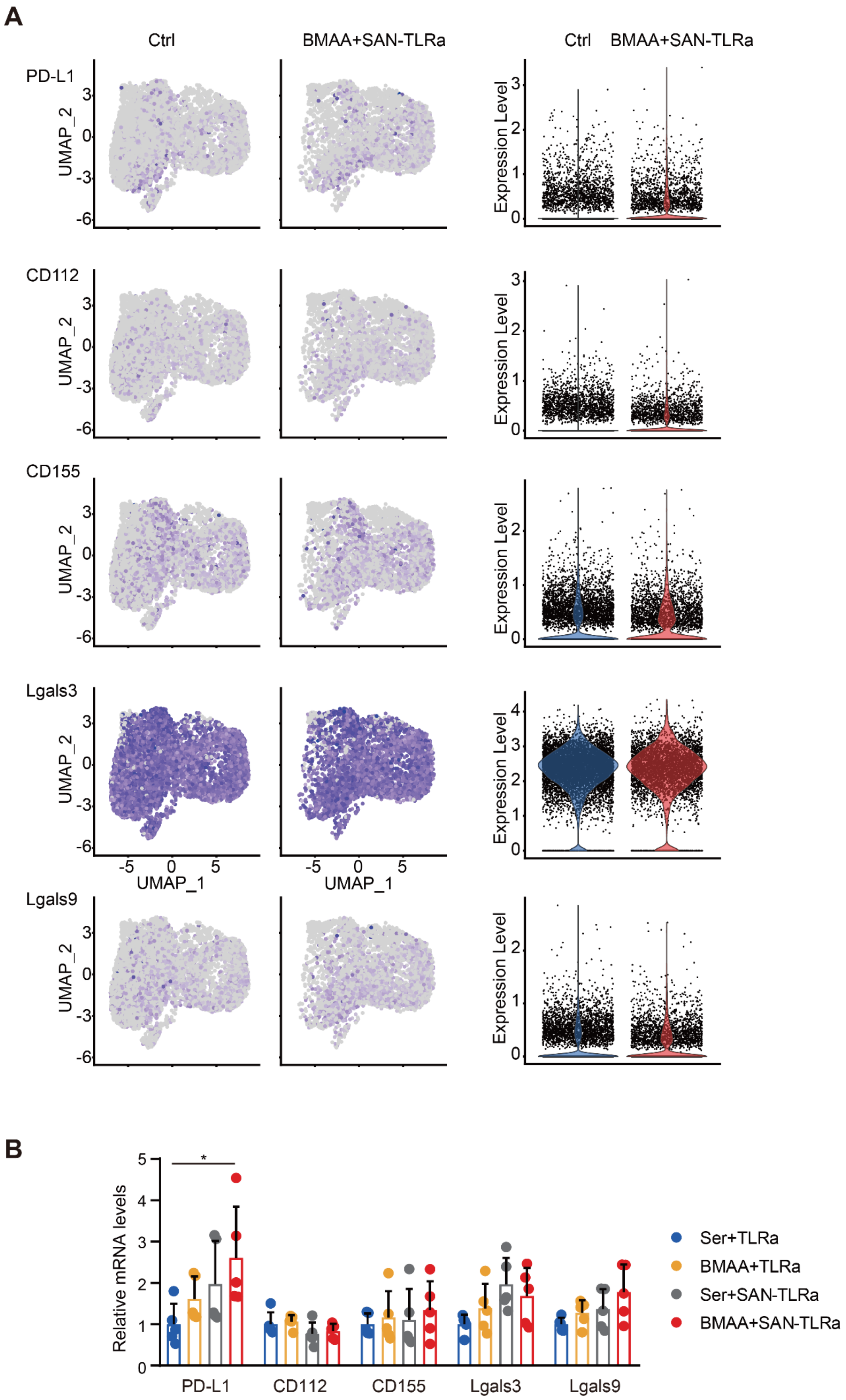
A
Ctrl
BMAA+SAN-TLRa
Ctrl
BMAA+SAN-TLRa
PD-L1
CD112
CD155
Lgals3
Lgals9
UMAP_2
UMAP_1
Expression Level
B
Relative mRNA levels
*
PD-L1
CD112
CD155
Lgals3
Lgals9
Ser+TLRa
BMAA+TLRa
Ser+SAN-TLRa
BMAA+SAN-TLRa

Figure S9 Immune checkpoint receptors in tumor were upregulated with BMAA combined with SAN vaccine. (**A**) Feature plots and violin plots of immune checkpoint receptor genes PD-L1, CD112, CD155, Lgals3, and Lgals9 in the mice receiving BMAA+SAN-TLRa or Ser+TLRa. Data analysis was conducted on the cancer cells in Figure 4B. (**B**) The expression of immune checkpoint receptor genes PD-L1, CD112, CD155, Lgals3, and Lgals9 in tumor tissues of mice with indicated treatments. The mice (n = 5) followed the same schedule as shown in Figure 3a. $^{*}P \leq 0.05$, *P* values were calculated by a one-way ANOVA test.

Table S1 The top 10 predicted MHC-I binding affinity of BALB/c mouse for peptides containing BMAA misincorporated sites

Allele	Protein	Length	Peptide	Score	Rank
H-2-L^d	Pola1	13	RPEYSDKSLYTQL	0.288724	0.23
H-2-L^d	Pola1	9	RPEYSDKSL	0.170392	0.47
H-2-L^d	Erap1	9	IPDFQSGAM	0.167546	0.47
H-2-K^d	Pola1	11	EYSDKSLYTQL	0.160977	0.49
H-2-L^d	Pola1	9	SDKSLYTQL	0.100334	0.77
H-2-L^d	Cse1l	9	LPGSSENEY	0.070424	1.1
H-2-L^d	Pola1	10	RPEYSDKSLY	0.060851	1.2
H-2-L^d	Pola1	10	YSDKSLYTQL	0.058411	1.2
H-2-L^d	Cse1l	11	LPGSSENEYIM	0.042712	1.6
H-2-K^d	Pola1	9	SDKSLYTQL	0.039474	1.7

Table S2 Routine blood and biochemical examination of non-tumor-bearing mice with indicated administration

	Ser+TLRa	BMAA+TLRa	Ser+SAN-TLRa	BMAA+SAN-TLRa	ANOVA
RBC (10^{12}/L)	5.99±0.04	6.12±0.18	6.39±0.02	6.12±0.07	ns
HGB (g/L)	127.67±1.45	129.67±5.55	137.67±1.2	134.33±1.86	ns
HCT (%)	22.07±0.23	22.97±0.93	23.8±0.1	22.87±0.22	ns
MCV (fL)	36.83±0.3	37.43±0.43	37.27±0.19	37.43±0.09	ns
MCH (pg)	21.33±0.12	21.17±0.29	21.53±0.13	22±0.1	ns
MCHC (g/L)	579.33±2.91	565±1.15	578±6.56	587±2.89	*
RDW-CV (%)	13.4±0.06	12.8±0.15	13.3±0	13.33±0.12	*
RDW-SD (fL)	20.83±0.23	20.17±0.43	20.8±0.1	21.03±0.23	ns
PLT (10^9/L)	428.67±10.35	491.67±33.45	382.33±59.34	342±25.24	ns
MPV (fL)	36.67±9.29	65±28.93	53.67±6.35	33.67±15.95	***
PDW	16.67±4.16	21±2.65	13±3.61	22.33±2.31	ns
PCT (%)	3.07±0.51	1.9±1.05	3.05±0.33	2.56±0.25	ns
TP (g/L)	47.75±1.05	49.7±3.24	49.77±0.32	49.13±0.44	ns
ALB (g/L)	27.17±0.7	28.13±1.42	29.03±0.93	29.27±0.62	ns
GLO (g/L)	20.93±1.03	21.57±1.92	20.73±0.69	19.87±0.35	ns
A/G	1.3±0.1	1.3±0.06	1.4±0.1	1.47±0.03	ns
TBIL (μmol/L)	3.48±0.84	3.3±0.84	3.13±0.83	3.36±0.37	ns
ALT (U/L)	77.33±12.6	80.67±29.28	64.67±0.88	62.33±1.45	ns
AST (U/L)	294.67±29.38	332.33±76.84	258.67±11.35	231±10.69	ns
AST/ALT	3.89±0.25	4.48±0.5	4±0.13	3.72±0.25	ns
GGT (U/L)	0.8±0.56	1.03±0.23	0.47±0.03	1.2±0.38	ns
TBA (μmol/L)	2.59±0.14	1.51±0.09	3.29±0.55	1.92±0.15	*

CK (U/L)	2286.67±474.86	2457.67±542.33	2000.67±197.59	1396.33±175.34	*
AMY (U/L)	834.67±222.93	941.33±283.45	800.67±37.96	658±10.15	ns
TG (mmol/L)	1.03±0.09	1.06±0.29	1.54±0.1	1.17±0.03	ns
CHOL (mmol/L)	4.47±0.35	4.39±0.77	4.73±0.09	4.66±0.23	ns
GLU (mmol/L)	7.54±0.84	9.04±2.01	7.7±0.81	7.17±0.53	ns
CRE (μmol/L)	68.67±7.31	46±5.51	38±2.08	61±15.72	ns
BUN (mmol/L)	9.88±1.28	11.54±1.91	8.17±0.15	7.2±0.4	ns
BUN/CRE	36.67±5.36	65±16.7	53.67±3.67	33.67±9.21	ns
tCO_2 (mmol/L)	16.67±2.4	21±1.53	13±2.08	22.33±1.33	*
P (mmol/L)	3.07±0.29	1.9±0.6	3.05±0.19	2.56±0.14	ns

Table S3 Primers and Antibodies used in this study

Primers for qRT-PCR		Sequences (5'-3')
Mouse Pola1	Forward	AGCCAACTTGTTGCAGTCGC
	Reverse	TTCCAGTGCACAGTCCGCAT
Mouse ACTB	Forward	TATCGCTGCGCTGGTCGTC
	Reverse	GGCCTCGTCACCCACATAGG
Mouse IFN-α	Forward	ATTTCCCCTGACCCAGGAAGATG
	Reverse	TTCCCAGCACATTGGCAGAGG
Mouse CD11c	Forward	AGCACACGGTTCTCCCTGAT
	Reverse	GCTGCCTTACAGAACCCAACA
Mouse CD86	Forward	ATGGACCCCAGATGCACCAT
	Reverse	ACTACCAGCTCACTCAGGCTT
Mouse PD-L1	Forward	GGCCGAGGGTTATCCAGAAG
	Reverse	AAACATCATTCGCTGTGGCG
Mouse CD112	Forward	ACGATCCAAAGACTCAGGTGT
	Reverse	CCGAGAGATGAGGGAGCCAT
Mouse CD155	Forward	ACACGGTCATTGTGTGCGAA
	Reverse	ATTGGACTGCAGAGCACGAC
Mouse Lgals3	Forward	ACCACTTCAAGGTTGCGGTC
	Reverse	GCGCTGGTGAGGGTTATGTC
Mouse Lgals9	Forward	ACCTTCCAGACTCAGAACTTTCGT
	Reverse	ATAGGCTGGGGTGGGGTACA

Antibodies for flow cytometry	Source	Cat
FITC MHC-II mAb	eBioscience	11-5321-82
PE CD335 mAb	eBioscience	12-3351-82
PE/Cyanine7 CD8a mAb	eBioscience	25-0081-81
PerCP/Cyanine5.5 CD11c mAb	eBioscience	45-0114-82
APC Foxp3 mAb	ebioscience	17-5773-80
FITC CD3 Antibody	BioLegend	100204
FITC CD11c Antibody	BioLegend	117306
FITC CD8a Antibody	BioLegend	100706
FITC Ly-6C Antibody	BioLegend	128006
FITC F4/80 Antibody	BioLegend	123108
PE CD45 Antibody	BioLegend	103106
PE CD206 Antibody	BioLegend	141706
PE CD86 Antibody	BioLegend	105008
PE CD4 Antibody	BioLegend	130310
PE Granzyme B Antibody	BioLegend	372208
PE/Cyanine7 Ly-6G Antibody	BioLegend	127618
PerCP/Cyanine5.5 I-A/I-E Antibody	BioLegend	107626
PerCP/Cyanine5.5 IFN-γ Antibody	BioLegend	505822

APC CD11b Antibody	BioLegend	101212
APC Granzyme B Antibody	BioLegend	396408
APC T-bet Antibody	BioLegend	644813
APC NK-1.1 Antibody	BioLegend	108710
APC CD80 Antibody	BioLegend	104714
APC CD45 Antibody	BioLegend	103112
APC/Cyanine7 CD11b Antibody	BioLegend	101226
APC/Cyanine7 CD3 Antibody	BioLegend	100222
FITC CD45R/B220 Antibody	BD	553087
PE IFN-γ Antibody	BD	554412
Antibodies for IHC	Source	Cat
Anti-mouse CD8α Rabbit mAb	CST	98941
Anti-Granzyme B Rabbit mAb	Abcam	ab255598
PD-1 Monoclonal Antibody	ThermoFisher	MA5-15780
PD-L1/CD274 Polyclonal antibody	Proteintech	17952-1-AP
HRP-conjugated Affinipure Goat Anti-Rabbit IgG	Proteintech	SA00001-2
HRP-conjugated Affinipure Goat Anti-Mouse IgG	Proteintech	SA00001-1

Supplementary Material 1 Single-Cell RNA Sequencing Reveals the Expression of Dendritic Cell Signature Genes in Tumor Tissue

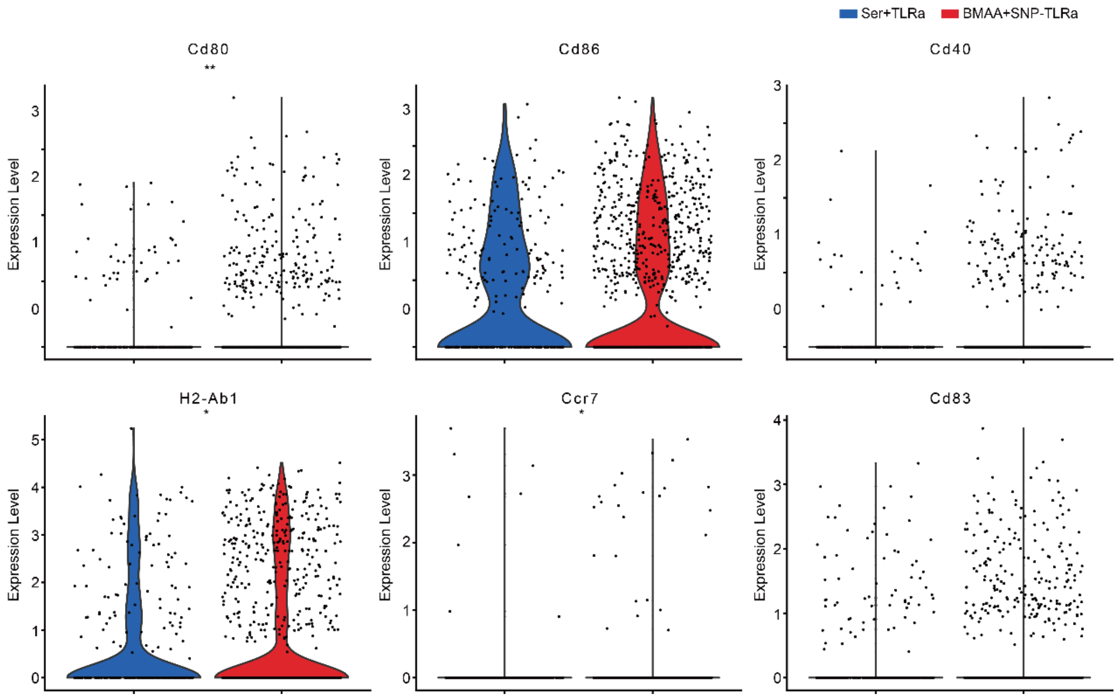

Supplementary Material 2 Single-Cell RNA Sequencing Reveals Subpopulations of $CD8^+$ and $CD4^+$ T Cells in Tumor Tissue

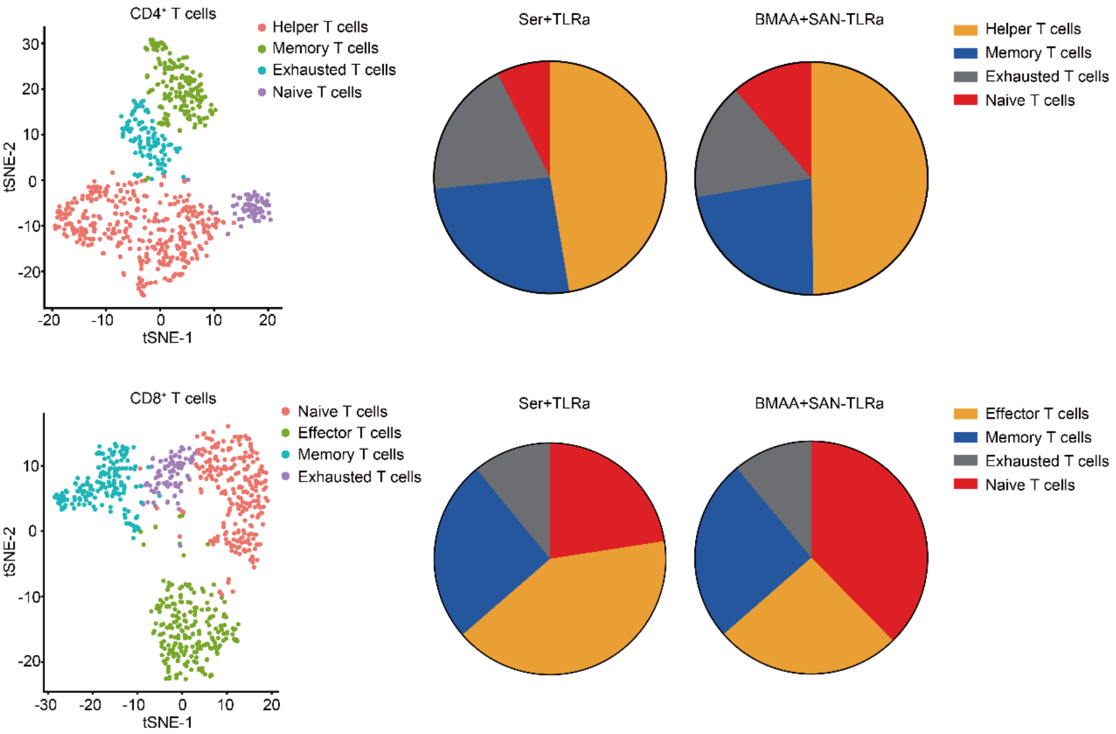

Supplement: Supplementary file 1 — Supplementary figures and tables. [file thnov15p3143s1.pdf]
